# Microhydration of Tertiary Amines: Robust Resonances in Red-Shifted Water

**DOI:** 10.1021/acs.jpclett.3c02517

**Published:** 2023-11-06

**Authors:** Eaindra Lwin, Taija L. Fischer, Martin A. Suhm

**Affiliations:** Institute of Physical Chemistry, University of Göttingen, Tammannstrasse 6, 37077 Göttingen, Germany

## Abstract

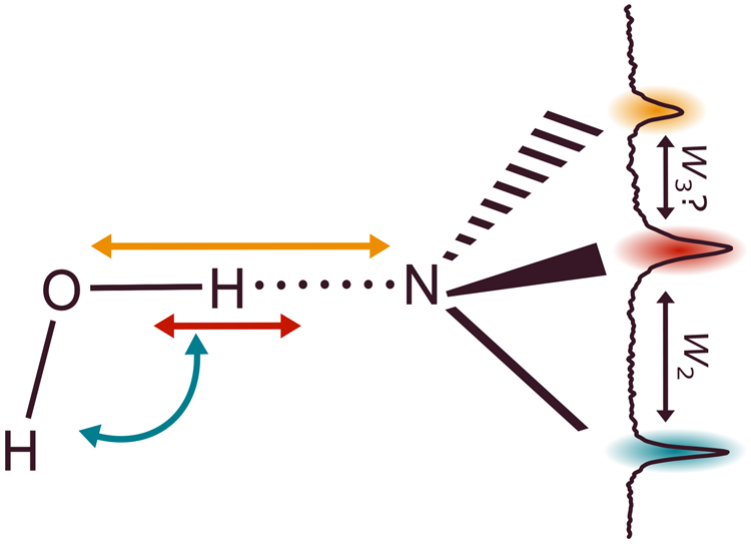

Tertiary amines are strong hydrogen bond acceptors. When a water molecule donates one of the OH groups, its in-phase stretching vibration wavenumber is decreased to such an extent that it comes close to the water bending overtone. This gives rise to anharmonic phenomena such as classical Fermi resonance, resonance with multiple-quantum dark states, or combination transitions with low-frequency intermolecular modes. These effects, which contribute to the spectral breadth of room-temperature hydrogen-bonded amine complexes, are disentangled by Fourier transform infrared spectroscopy in pulsed supersonic slit jet expansions. Monohydrates of the amines quinuclidine, *N*-methylpyrrolidine, *N*-methylpiperidine, and dimethylcyclohexylamine exhibit systematic mode coupling signatures. These suggest relatively fast energy flow out of the excited OH stretching fundamental into intra- and intermolecular degrees of freedom of the hydrogen-bonded water molecule. Trimeric complexes are spectroscopically separated from the amine monohydrates.

The energy deposited in OH stretching vibrations of isolated water molecules in the electronic ground state will stay there for quite some time,^1,2^ because neither rotational nor OH bending degrees of freedom offer doorway quantum states for near-resonant vibrational redistribution, quite in contrast to larger OH-containing molecules.^3,4^ This changes when the water molecules aggregate, because the hydrogen bonding brings stretching fundamentals and the first bending overtones closer together.^5−7^ In the frequency-domain vibrational spectrum, the onset of resonance manifests itself as a transfer of spectral intensity from the stretching transitions to the bending overtones or to states built on these bending overtones. Direct and strong stretch–bend Fermi resonance has previously been assigned in the Raman spectrum of jet-cooled water clusters larger than dimers.^8^

A more subtle resonance variant can even be observed in dimeric complexes. If the acceptor water molecule is replaced by a ketone,^9^ the donor water binds more strongly. This can bring the hydrogen-bonded OH stretching fundamental of the donor into the vicinity of a three-quantum excitation of bend overtone and water libration in the 1:1 complex,^9^ with a coupling matrix element on the order of 10 cm^–1^. It appears logical to extend this concept to even stronger isolated hydrogen bonds, which render the bridging librational quantum obsolete and allow for a direct Fermi resonance between the hydrogen-bonded stretch and the bending overtone of the librating water unit. In this work, we show in a spectroscopically resolved way that this indeed happens systematically when water binds to tertiary amines. We also show that the low-frequency intermolecular motion of the water molecule interferes with the dominant intramolecular resonance. Surprisingly, this does not happen in the form of familiar Franck–Condon-like progressions on top of the hydrogen-bonded OH stretch, but instead on top of the initially dark bend overtone resonance partner, perhaps through more indirect^10^ coupling mechanisms. These infrared spectroscopic signatures of anharmonic motion in a strongly solvating single water molecule invite accurate modeling of the nuclear dynamics that is behind the spectral complexity.

The tertiary amines that we investigate in this work may be described by enumerating the isolated alkyl substituents (M for methyl and C for cyclohexyl) attached to the N atom. If these substituents are linked into rings to reduce flexibility and to introduce strain into the N coordination, the size of the hydrocarbon bridge follows the letter N. Specifically (see Figure 1), we investigate (in order of decreasing strain) N555 (quinuclidine or 1-azabicyclooctane), MN4 (*N*-methylpyrrolidine), MN5 (*N*-methylpiperidine), and MMCN (dimethylcyclohexylamine), which all feature at least one symmetry plane. These four amines are co-expanded with varying amounts of water in a large excess of inert carrier gas through a long (0.7 m) and thin (0.2 mm) slit nozzle during pulsed operation. The OH stretching spectrum between the free water and CH stretching region is recorded by synchronized Fourier transform infrared (FTIR) spectrometer scans. References (9) and (11) contain more details. In the work presented here, we use a helium expansion pressure on the order of 750 hPa and a high (on the order of 1:1:1000) dilution of the amine and water to obtain rotationally and vibrationally cold dimers, essentially free of contributions from larger clusters. For comparison, we also investigate an ultrasoft (20 hPa) co-expansion at a low (0.5:0.5:1) dilution in neon for the volatile amine MN4, to reveal some hot band contributions that will dominate the room-temperature spectra of complexes.

**Figure 1:**
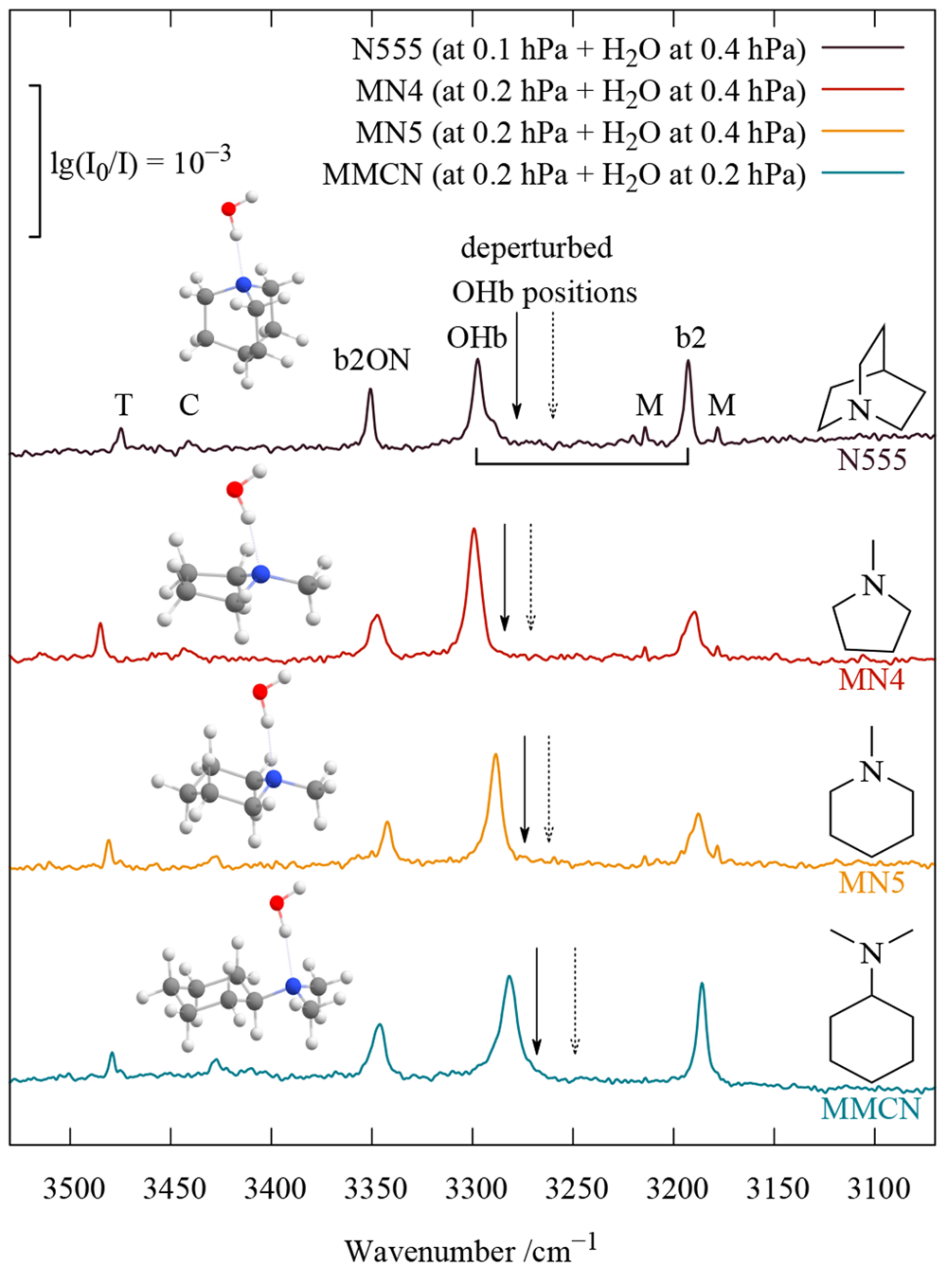
Hydrogen-bonded OH stretching infrared spectra of jet co-expansions of four amines with water, showing monohydrate water vibration triplets that are assigned to the bright OHb transition in resonance with bend overtone b2 and tentatively with b2 in combination with dimer stretching (ON) or some other low-frequency mode. Weak features from trimers (T), clusters of unclear size (C), and the water monomer (M, bend overtone) are also marked. Dashed and continuous arrows refer to deperturbed OHb positions, based on the intensities and positions of two and three experimental signals, respectively. See the text for further details.

The cold spectra (Figure 1) show a striking similarity in the region around 3300 cm^–1^ where the unperturbed hydrogen-bonded OH stretching fundamental (OHb) would normally be expected with a single signal. Instead, a strong central band is flanked by two more narrow and weaker satellites. The central band has some fine structure, most prominently for N555, which we assume to be due to subtle resonances, but contributions from larger clusters cannot always be strictly ruled out. The lower-frequency satellite is downshifted by ∼100 cm^–1^ (illustrated by a bracket) and falls in the range where the overtone of the water bending vibration (b2) is located. Its intensity strongly exceeds expectation for an infrared overtone, as indicated by the tiny bend overtone signals of the water monomer (M) that is present in large excess. This points to a resonance situation in which the OHb mode shares some of its infrared intensity due to wave function mixing.^12^ The associated coupling constant is denoted *W*_2_. The higher-frequency satellite of similar intensity in Figure 1 is shifted by only approximately half the amount from the central OHb transition. If it also derives its intensity from anharmonic mixing with OHb, the extent of coupling (*W*_3_) must be reduced compared to that of direct OHb–b2 coupling. A plausible assignment is b2 in combination with some intermolecular mode, as in the case of ketone monohydrates.^9^ Due to the much larger downshift of OHb in amine hydrates, it will not be a high-frequency librational mode^13^ (Lip, Lop, *vide infra*), but rather some low-frequency displacement of the water molecule relative to the amine. Given the spacing between the two satellite modes of ≲150 cm^–1^ (after deperturbation), there are two plausible candidates. One is the overtone (t2) of the torsion (t) of the free OH group around the hydrogen bond. This is a very soft^14^ and anharmonic, nearly free internal rotation. Its overtone matches the symmetry of OHb and b2, even if the complex minimum structure is approximately symmetric with respect to the plane of the water molecule. The other candidate is the hydrogen bond or O···N stretching fundamental^15^ (ON), which is also quite anharmonic, at least in the related water dimer case.^16^ It is not clear whether this mode stiffens or weakens in combination with b2;^17^ however, probably the diagonal anharmonicity dominates, and the b2ON–b2 difference should thus fall below its harmonic prediction. Experimentally, it currently has to remain open which of these (b2t2, b2ON, or any other) intra-intermolecular candidates for coupling gives rise to the third spectral feature, whose relative and absolute position is quite independent of the specific amine. ON stretching (such as b2) might be less dependent on the substitution pattern of the amine than a torsional overtone. In the reference case of the water dimer,^18^ the torsion overtone and the dimer stretch are possibly mixed and a specific assignment may thus not be trivial in amine hydrates, either. Still, encouraged by the regular spectral pattern of the four amines despite rather different low-energy torsional landscapes (Figures S1–S5), we tentatively assume that b2ON is an appropriate descriptor for the high-frequency component of the resonance triad in amine hydrates.

At this stage, one should not dismiss an alternative interpretation of the spectral triplet, where the OHb–b2 Fermi resonance explains the intensity of the lowest-frequency transition but the higher-frequency component is a combination transition building on OHb, rather than a resonant state building on b2. In this case, the symmetry of the intermolecular excitation could also be non-totally symmetric, such as for t, which cannot undergo a resonance with OHb or b2. Because the OHb character is partially transferred to b2 through the Fermi resonance, there should then be a weaker corresponding b2 combination visible in the spectrum, as well. As this is largely not the case in the four investigated amines (except for very weak features, e.g., in MN4 or MMCN), we currently favor a resonance triad (b2NO, OHb, and b2) as the explanation for the observed spectral pattern. The other possibility that the highest-frequency partner of the triplet builds on an amine vibration rather than a water vibration appears to be quite remote due to its persistence in all four amine monohydrates.

To back some of this empirical reasoning, Figure 2 shows harmonic DFT (B3LYP-D3/def2-TZVP^19,20^ and ORCA 5.03^21^) predictions of the low-frequency modes for all four systems. The six levels that arise from complexation are shown as full lines (two soft out-of-plane and in-plane bendings, op and ip, respectively, free OH torsion t, the intermonomer ON stretch, and two stiff in-plane and out-of-plane librations, Lip and Lop, respectively, which are responsible for the b2lib resonance^9^) connected by colored dashes. Black dashed lines represent amine fingerprint modes in both the monomer (left) and the complex (right). In some instances, there is harmonic mode mixing that gives partial intermonomer character to some of the amine modes (in which case only the monomer position is dashed). The colored connecting lines support the insensitivity of ON, but also t, to chemical substitution. This leaves open the interpretation of the upper resonance partner as b2ON or b2t2, but for the sake of simplicity, we settle for the somewhat more plausible ON interpretation in the following, also given the approximately quadratic energy dependence on the quantum number of a nearly free torsion.

**Figure 2:**
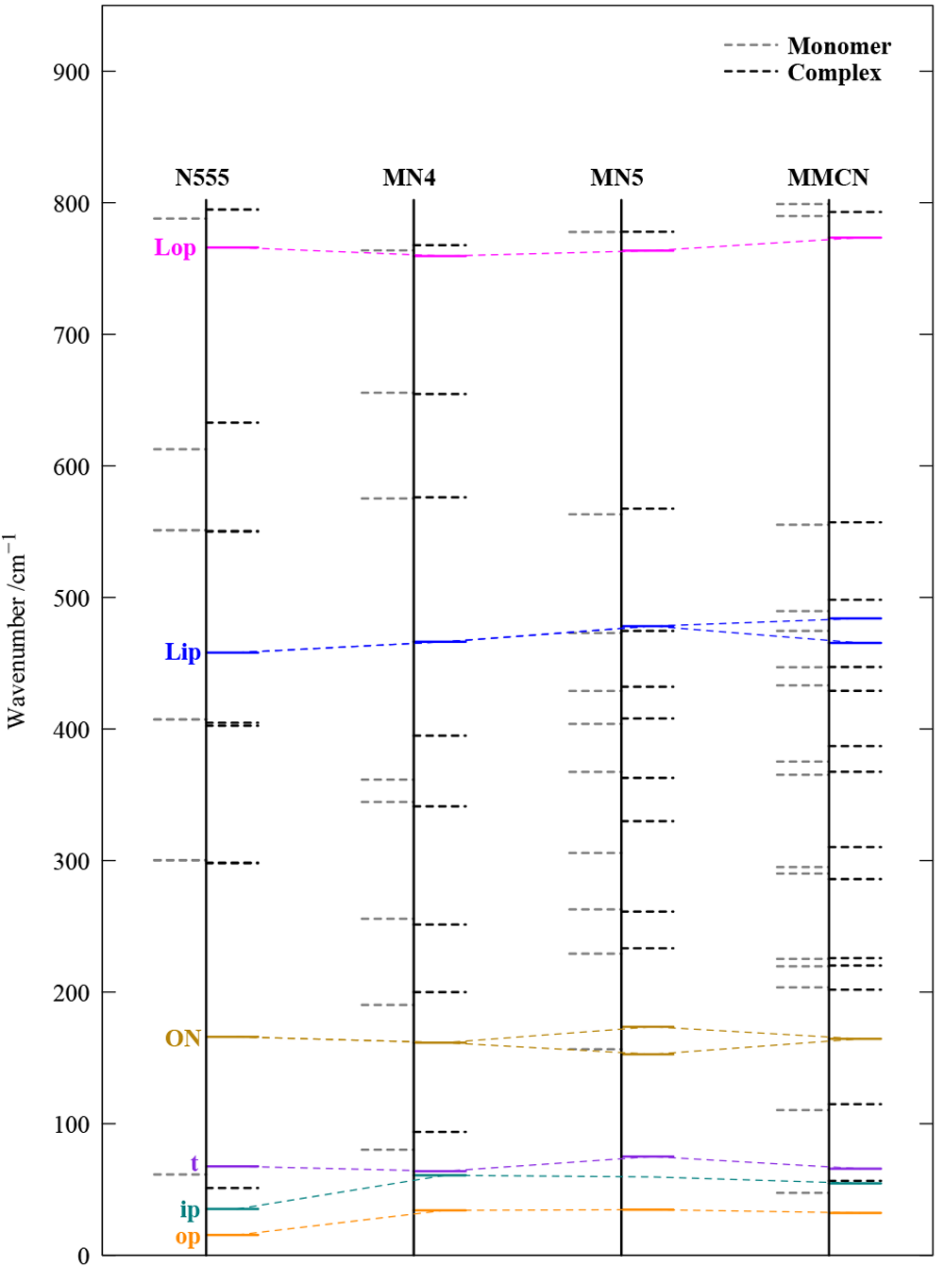
Harmonic B3LYP-D3/def2-TZVP predictions of the six newly generated vibrations in the monohydrates (full colored lines), embedded in amine modes (dashed) for the monomers (left) and their complexes (right). Where intra- and intermolecular modes come close, harmonic mixing may occur, leading to a mixed character and shifted positions, as indicated by bifurcated (colored) connecting lines among the four amine columns.

On a largely experimental basis, the comparison of the four amine monohydrates in Figure 1 thus suggests that there is a uniform pair of dark states b2 and b2ON surrounding OHb, stealing IR intensity from it and shifting it up (b2) and down (b2ON) in energy. The black arrows in Figure 1 mark the position of the deperturbed OHb band if both perturbers derive their intensity from OHb; however, the dashed arrow marks the position if only b2 is resonance active, and the upper state is instead a combination band with OHb carrying intrinsic oscillator strength, such as OHbt. This forms an excellent playground for anharmonic models of hydrogen-bonded water molecules, bridging the regular dynamics of weaker hydrogen bonds of water, e.g., to alcohols^22^ or ketones^9^ and the heavily coupled dynamics of carboxylic acid complexes.^23^ It also defines an interesting challenge for the reliable prediction^22^ of OHb frequencies when the hydrogen bonding becomes stronger. Other than for phenol–amine complexes that can be accessed via truly size-selective methods,^24^ there is a clear separation of the OH vibrations from CH vibrations in the 1:1 complexes, which helps in the assignment.

While for the more weakly coupled water dimer, there are extensive ab initio-based theoretical models of OHb frequencies ranging from reduced^25^ to full dimensionality,^26^ anharmonic models are more sparse for the more strongly coupled neutral amine hydrates.^15,27^ In preparation of such a first-principles theoretical analysis for the systems investigated in this work, we shall discuss a set of simple empirical coupling models, based on effective coupling constants *W*_*n*_ between the bright OHb state and *n*-quantum intra/intermolecular excitations that are assumed to be spectrally dark without resonance. In this limit, the integrated intensity of the observed transitions (including any fine structure on the wings), obtained by different variants of a stochastic method,^28^ allows the derivation of the extent of mixing of the interacting states. The simplest model (A) assumes that there is only a two-level Fermi resonance between OHb and b2, whereas any combination bands building on b2 (or OHb) are omitted from the analysis. This leads to a single coupling constant, *W*_2_, based on the observed relative intensity of OHb and b2. Ignoring significant IR intensity at higher wavenumbers, this model provides a realistic lower bound of the deperturbed OHb position. A sequential coupling model (model B) first perturbs the interaction of the presumably triple-quantum combination band with the OHb fundamental (implying a coupling constant *W*_3_) and then further decouples the resulting state (with combined intensities of b2ON and OHb) from the b2 resonance to obtain *W*_2_. The resulting deperturbed OHb position corresponds to the center of gravity of the three transitions (marked with a solid arrow in Figure 1), as would the opposite sequence of deperturbations (deperturbing OHb first from b2 and then from b2ON). Simultaneous treatment of all three states requires further assumptions about direct coupling between b2 and b2ON that will be explored in the future. Rewardingly, the standard deviation of the deviations of model A (dashed arrows in Figure 1) or B (full arrows in Figure 1) from a scaled harmonic harmonic prediction of OHb (last column of Table 1, B3LYP-D3/def2-TZVP) is approximately half of that of the raw positions of the dominant experimental peak (with model A performing slightly better), suggesting that spectral deperturbation helps to meet harmonic theory.

**Table 1:** Deperturbed OHb Band Positions and Effective Resonance Coupling Parameters (in cm^–1^) for Amine Monohydrates from Models A and B Compared to the Raw Spectroscopic Data (dominant peak positions) and to Scaled Harmonic Prediction 0.97ω

	raw	A	B	0.97ω
N555				
OHb	3298	3260	3278	3265
*W*_2_ (b2)	–	51	55	
*W*_3_ (b2ON)	–	0	24	
MN4				
OHb	3299	3271	3284	3282
*W*_2_ (b2)	–	48	49	
*W*_3_ (b2ON)	–	0	21	
MN5				
OHb	3289	3262	3274	3268
*W*_2_ (b2)	–	45	47	
*W*_3_ (b2ON)	–	0	21	
MMCN				
OHb	3282	3249	3268	3259
*W*_2_ (b2)	–	46	51	
*W*_3_ (b2ON)	–	0	28	

The coupling models involving two (A) or three (sequential or simultaneous) states are illustrated in the resonance scheme shown in Figure 3. The observed transitions in the center can be deperturbed to provide zero-order states ()_0_ without the effect of anharmonic resonance. Depending on whether the third state is included in the analysis, (OHb)_0_ and (b2)_0_ are more or less degenerate, and further work such as isotope substitution, compound extension, and Raman spectroscopy will be required to make a decision between the models.

**Figure 3:**
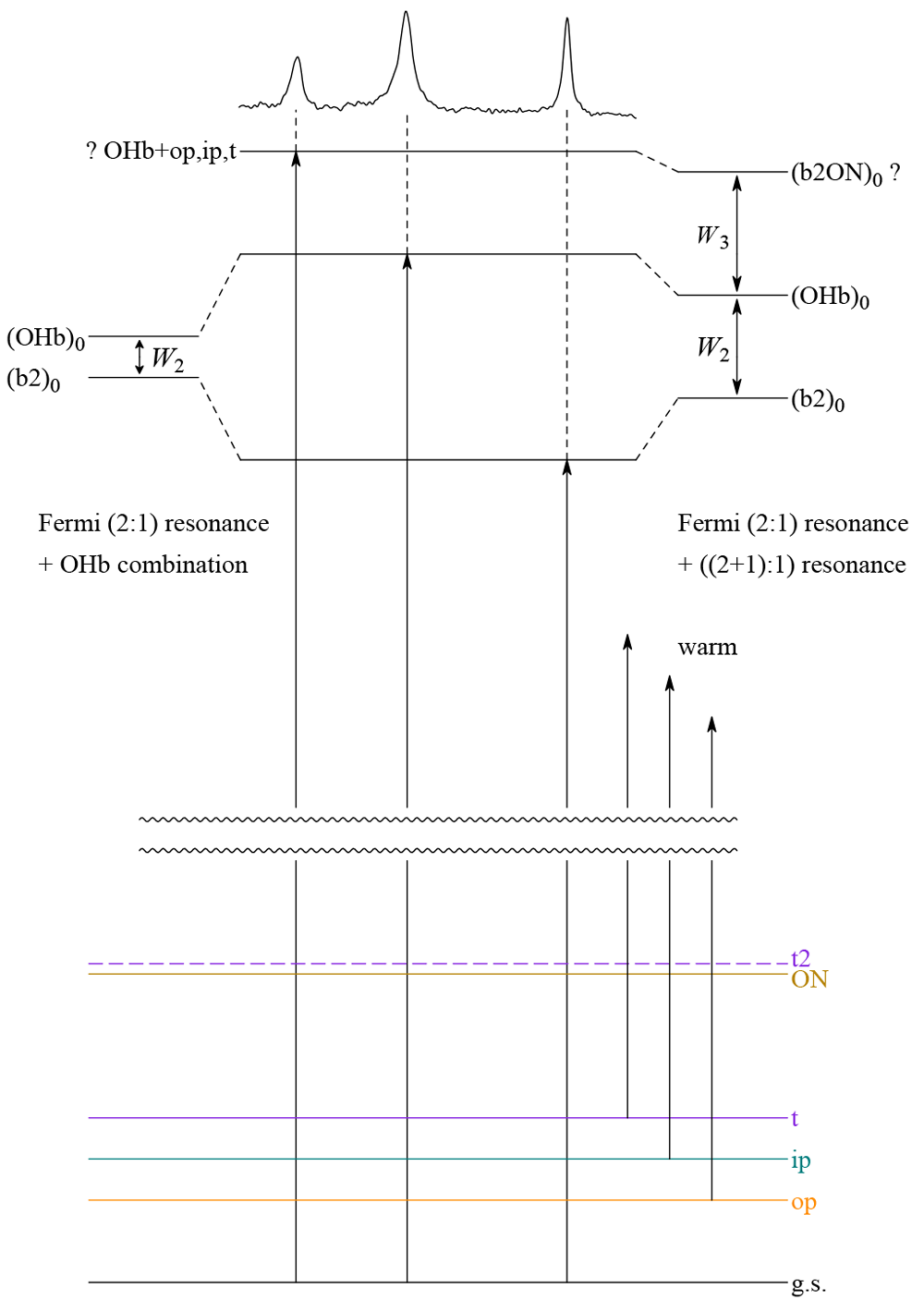
Qualitative energy level scheme for coupling models A (left) and B (right), which distribute the zero-order OHb stretching character among different intra- and intermolecular states of amine monohydrates. See the text for explanations.

Because our jet FTIR spectra include all cluster sizes present in the expansion, variation of the conditions is helpful to safely establish which signals are really due to 1:1 complexes of water (W) and an amine (A) and which are perhaps due to 1:2 or 2:1 trimers. The extreme dilution used in Figure 1 down to 1:1:3750 W:A:He gives such trimers a low abundance. This is shown by comparison to higher concentrations for volatile amine MN4 in Figure 4, now also extending into the water monomer stretching range (M), where the out-of-phase or free OH vibration (OHf) is visible.

**Figure 4:**
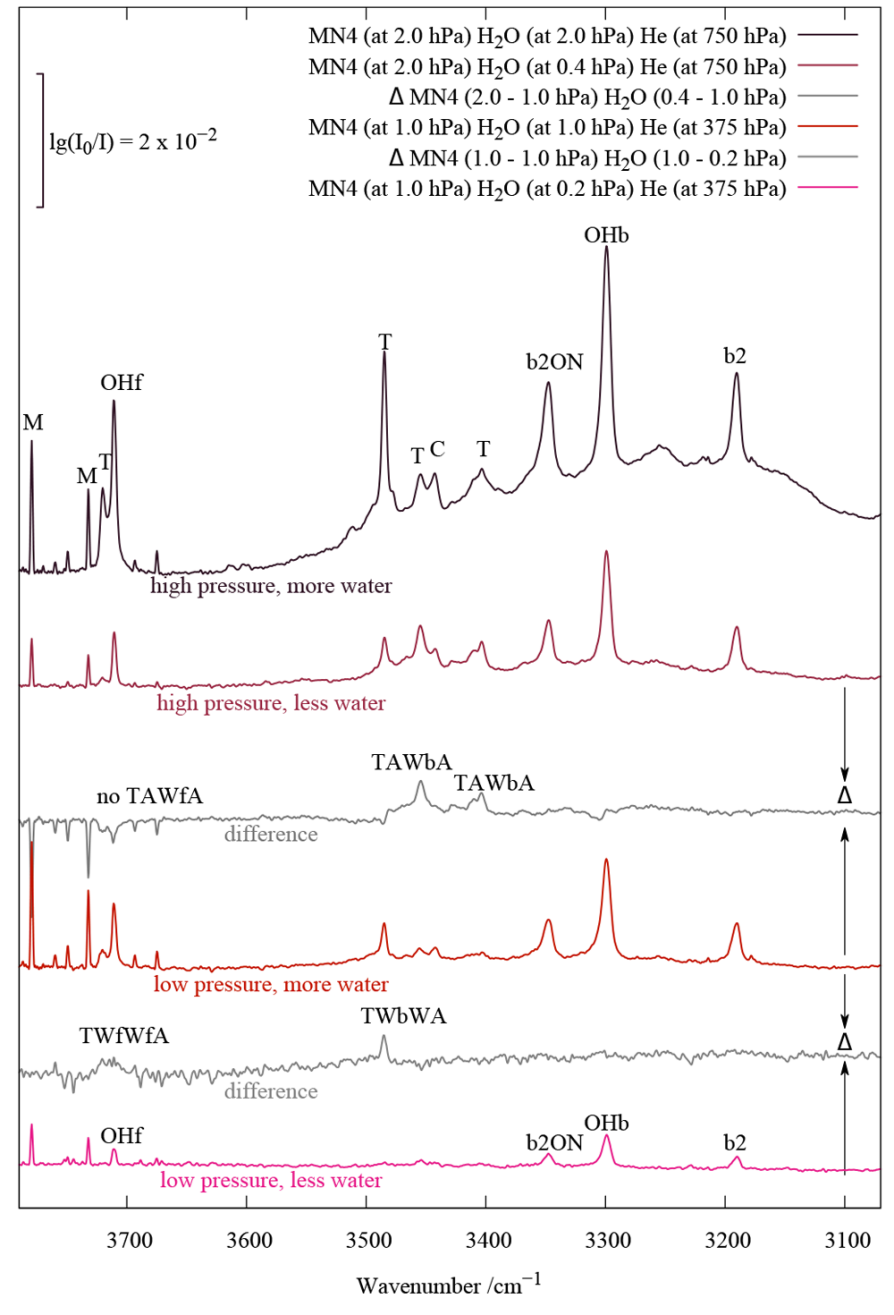
W–A co-expansions at different stagnation pressures and concentration ratios in the case of A = MN4, which can be used for selective cancellation of certain cluster sizes by spectral subtraction (Δ traces between adjacent original spectra). WWA and AWA trimer contributions can be cleanly separated from the dominant WA dimer features. See the text for further explanations.

Specifically, 5:5 and 1:5 compositions (W:A) were investigated at two different stagnation pressures. Spectral subtraction at low pressure to remove the mixed dimer contributions reveals a narrow water-rich trimer signal at a wavenumber lower than that of the water dimer (TWbWA, water dimer terminated by an amine) and a broad feature (TWfWfA), partially upshifted from OHf of the monohydrate, due to the two dangling OH groups of this WWA trimer. Spectral subtraction between the high-pressure 1:5 expansion and the low-pressure 5:5 expansion simultaneously cancels the mixed dimer and the WWA trimer to a reasonable extent (leaving some baseline wiggles due to less vibrational cooling at a lower stagnation pressure). It reveals two structured bands due to water hydrogen bonding in amine-rich trimers (AWA), but no positive free OH contribution. Clearly, the two amine units bind anticooperatively to the water (leading to a frequency upshift compared to the mixed dimer and qualitatively recovering the symmetric–antisymmetric pattern known for the water monomer, at half of the monomer splitting), which differs from the case for primary amines.^12^

Figure 4 thus unambiguously shows that the spectral OH signature of the mixed WA dimer at low temperatures is restricted to the three features discussed extensively. This may cast some doubt on recent reports of a much richer spectral OH signature assigned to the monohydrate of trimethylamine in a VUV-IR study.^15^ Some of the signals observed there might actually be due to mixed trimers, similar to the situation for water clusters.^9,22^ This invites a direct comparison of the two techniques for the same species in future work. One should also revisit an interpretation of matrix-isolated water – trimethylamine spectra,^29^ which invoked a cluster of two amine and four water units to explain a triplet similar to the one observed in this work, together with some absorptions in the CH region. We think that the experimental OH stretching signature of cold amine monohydrates may be less complex than suggested by previous experiments.^15,29^

Further anharmonic information can be obtained by moving away from the cold expansions shown in Figure 1, toward expansions that come closer to the room-temperature gas-phase spectrum^27^ but still ensure some cooling and sufficient complexation. This is illustrated for MN4 in Figure 5 by comparing a 1:2:3750 expansion in helium at 750 hPa to a 1:1:2 expansion in neon at a total stagnation pressure of 20 hPa. One can see how the three resonance peaks broaden asymmetrically, toward the monomer OH transition at 3657 cm^–1^, due to transitions from [mostly ip- and op- (see Figure 2)] vibrationally excited states that weaken the hydrogen bond. New transitions appearing to the left and right of the b2 signal may be due to hot, difference, or combination transitions involving the very low-frequency modes (op, ip, and t) and partially profiting from Fermi resonance to their OHb counterparts, but this requires a systematic investigation for several amines. Note that as the temperature increases and the hydrogen bond weakens, the relative intensity of the b2ON combination transition also increases, suggesting that it participates in the intensity stealing from the thermally blue-shifting OHb.

**Figure 5:**
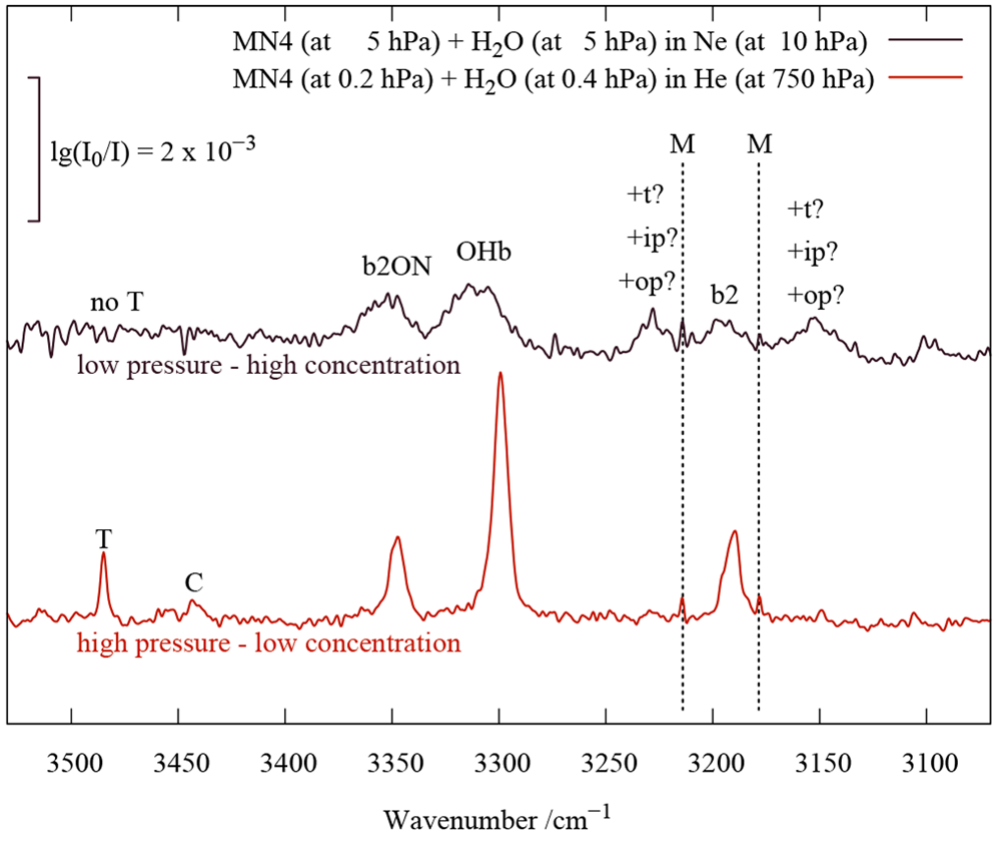
Comparison of a cold expansion of MN4 and H_2_O in He (red) to a very soft expansion in Ne (black), which gives rise to numerous hot transitions involving excited intermolecular modes (see also the scheme in Figure 3). These tend to reduce the downshift of vibrations with OH stretch character due to a weakening of the hydrogen bond, and they are seen to strongly intensify some bands in the vicinity of b2. Trimers do not form under these conditions.

In summary, tertiary amines activate the Fermi resonance between the hydrogen-bonded stretching fundamental and bending overtone in a first solvating water. They also lead to a third systematic spectral feature that likely involves a combined inter-intramolecular excitation, which surprisingly builds on the dark intramolecular state (Figure 6), and that contributes to the spectral width of room-temperature spectra of such strong hydrogen bonds.^27^ By inclusion of isotopologues, extension to other tertiary amines and further nitrogen compounds,^30^ application of more refined coupling models, and systematic generation of warm expansions, we hope to further clarify the vibrational resonance pattern of amine hydrates and to more accurately locate the zero-order OH stretching transition, which is of interest for the benchmarking of vibrational dynamics calculations.

**Figure 6:**
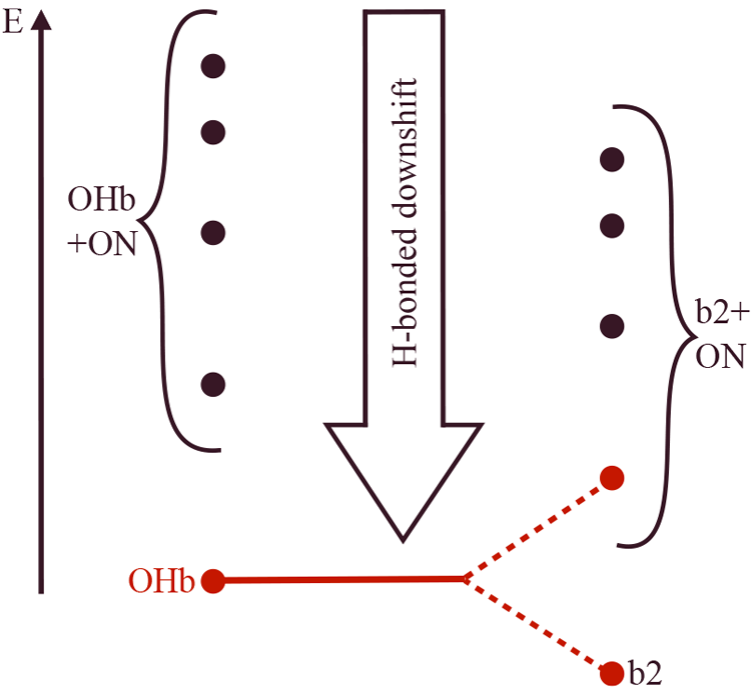
In monohydrates of tertiary amines, the hydrogen-bonded and thus downshifted OHb stretching mode does not light up intermolecular ON combination bands on top of OHb (left) but rather on top of the Fermi-resonant bending overtone b2 (right, red dashes symbolizing redistribution of OHb infrared intensity).

## Supporting Information Available

Experimental details, integration methods, quantum chemical calculations, and resonance models (PDF)
Transparent Peer Review report available (PDF)
