# Supplementary material for: Microhydration of Tertiary Amines: Robust Resonances in Red-Shifted Water
Source: J Phys Chem Lett. 2023 Nov 6;14(45):10194–9. doi: 10.1021/acs.jpclett.3c02517 (PMC10658632; doi:10.1021/acs.jpclett.3c02517)
Supplement: Supplementary file 1 — jz3c02517_si_001.pdf [file jz3c02517_si_001.pdf]

Microhydration of Tertiary Amines: Robust Resonances in Red-shifted Water

Eaindra Lwin, Taija L. Fischer, Martin A. Suhm*

Institute of Physical Chemistry, University of Göttingen, Tammannstr. 6, 37077 Göttingen (Germany). E-mail: msuhm@gwdg.de

Contents

1 Experiment **S3**
- 1.1 Investigated compounds . . . S3
- 1.2 Experimental details . . . S3

2 Harmonic modeling **S3**
- 2.1 Details on harmonic calculations . . . S3
- 2.2 Optimized structures of 1:1 amine water complexes . . . S5
- 2.3 Torsional scans . . . S8
- 2.4 Example input . . . S12

3 Integration methods **S13**
- 3.1 Method I . . . S13
- 3.2 Method II . . . S13
- 3.3 Method III . . . S13
- 3.4 Method IV . . . S13
- 3.5 Average intensity fraction . . . S13

4 Simple 3-state resonance models **S13**
- 4.1 Model A . . . S13
- 4.2 Model B . . . S14
- 4.3 Exemplary sensitivity analysis . . . S15

5 Experimental wavenumbers and scaled harmonic predictions **S16**

References **S16**

List of Tables

- S1 Table of investigated compounds . . . S3
- S2 Experimental details for the figures in the main text . . . S3
- S3 Lower wavenumber amine modes for monomers and monohydrates . . . S4
- S4 xyz coordinates of N555 . . . S5
- S5 xyz coordinates of MN4 . . . S6
- S6 xyz coordinates of MN5 . . . S6
- S7 xyz coordinates of MMCN . . . S7
- S8 Relative energy for different amine conformers using B3LYP-D3/ma-def2-TZVP . . . S8
- S9 Relative energy for different amine conformers using B3LYP-D3/def2-TZVP . . . S8
- S10 Example input . . . S12
- S11 Experimental intensity ratio . . . S14
- S12 Deperturbed peak position of OHb and coupling constants . . . S15
- S13 Deperturbed peak position of OHb and coupling constants sensitivity analysis . . . S15
- S14 Comparison of experimental and scaled theory predictions . . . S16

List of Figures

- S1 Torsional scan for N555+water . . . S9

S2 Torsional scan for MN4+water . . . S9
S3 Torsional scan for MN5+water . . . S10
S4 Torsional scan for MMCN+water . . . S10
S5 Comparison of all four torsional scans using B3LYP-D3/ma-def2-TZVP . . . S11
S6 Comparison of all four torsional scans using B3LYP-D3/def2-TZVP . . . S11

1 Experiment

1.1 Investigated compounds

Tab. S1 gives detailed information about the chemicals used for the study and introduces the codes for the chemical names that are also used in the main document.

Table S1: Table of investigated chemicals, the introduced code names (supplement and main document), their CAS number, the supplier, purity and Lot#.

Name	Code	CAS Number	Supplier	Purity	Lot#
1-Azabicyclooctane	N555	100-76-5	BLDpharm	98%	DLX952
N-Methylpyrrolidine	MN4	120-94-5	TCI	98%	QV5SL-KH
N-Methylpiperidine	MN5	626-67-5	TCI	99%	BK82C-SY
Dimethylcyclohexylamine	MMCN	98-94-2	TCI	98%	A6M8I-OH
Helium	-	7440-59-7	Nippon	99.996%	-
Neon	-	7440-59-7	Linde	99.999%	-

1.2 Experimental details

The experimental setup for the FTIR measurements is introduced in great detail in [1] and more information on the recorded spectra can be found in Tab. S2.

Table S2: Spectroscopic details of the figures 1, 4 and 5 in the main document. The partial pressures of the amine (p_A), water (p_w), as well as the carrier gases helium (p_{He}) and neon (p_{Ne}) and the total stagnation pressure of the expansion (p_s) are given. The spectra were obtained by averaging # FTIR scans during a 133 ms gas pulse through a 700 mm×0.2 mm slit nozzle with a Bruker VERTEX 70v FTIR spectrometer in double-sided mode at 140 kHz scanning speed. A 20 W tungsten light source, an InSb/HgCdTe sandwich detector and an optical filter (wavenumber range <4000 cm^{-1}) were used. The date of the spectrum being recorded is given in a dd/mm/yyyy format and in the last column it is indicated in which figure in the main publication the corresponding spectrum is shown.

p_A/hPa	p_w/hPa	p_{He}/hPa	p_{Ne}/hPa	p_s/hPa	#	dd/mm/yyyy	Figure
N555							
0.1	0.4	750	0	750	800	12/06/2023	1
MN4							
0.2	0.4	750	0	750	1000	27/07/2023	1
2.0	2.0	750	0	750	200	01/08/2023	4
2.0	0.4	750	0	750	250	28/07/2023	4
1.0	1.0	375	0	375	200	01/08/2023	4
1.0	0.2	375	0	375	200	28/07/2023	4
5.0	5.0	0	10	20	1000	15/08/2023	5
MN5							
0.2	0.4	750	0	750	1000	28/06/2023	1
MMCN							
0.2	0.2	750	0	750	1000	04/07/2023	1

2 Harmonic modeling

2.1 Details on harmonic calculations

The molecular structures of N555, MN4, MN5 and MMCN were optimized by using three-body-inclusive D3-dispersion-corrected[2,3] B3LYP with the def2-TZVP basis set[4–6] (B3LYP/TZ) on Orca 5.0.3[7–10] with the keywords ABC DEFGRID3 VERYTIGHTSCF VERYTIGHTOPT FREQ. One water was then attached to the monomers using Chemcraft version 1.8 and the generated monohydrates were preoptimized using Crest[11,12]. After that, the 2-4 lowest energy structures were reoptimized using B3LYP-D3/def2-TZVP on Orca 5.0.3 with the same keywords used for the monomers. The trimer stuctures built from N555+2H_2O, MN4+2H_2O, 2MN4+H_2O, MN5+2H_2O and MMCN+2H_2O were preoptimized by using Crest and the resulting structures were reoptimized using the same functional and basis set as for monomers and mixed dimers on Orca 5.0.3.

Table S3: Harmonic DFT predictions on B3LYP/TZ level of theory of low wavenumber modes for the four investigated amines (monomers and monohydrates). The monohydrate modes with intermolecular motion character which may modulate the water Fermi resonance or appear as OHb combination bands are marked with different colors and labeled in the empty monomer field with an arrow. The soft in plane (ip) bending motion is given in green and the out of plane (op) bending motion in orange, the torsion (t) of the free OH of the water is shown in violet, the movement changing the distance between water and amine (ON) is depicted in brown, the in plane libration (Lip) is given in blue and the out of plane libration (Lop) in magenta. The other vibrations (black) are amine-specific (see Fig. 2 in the main text). If an intermolecular mode harmonically mixes with an amine mode, both are labeled in the corresponding color in both rows.

N555		MN4		MN5		MMCN	
Monomer	Complex	Monomer	Complex	Monomer	Complex	Monomer	Complex
op →	15	op →	34	op →	35	op →	32
ip →	35	ip →	61	ip →	60	ip →	55
61	51	t →	64	t →	75	47	56
t →	68	80	94	ON →	153	t →	66
ON →	166	ON →	162	ON →	174	110	115
300	298	190	200	157	233	ON →	164
300	298	256	251	229	261	204	202
407	403	345	341	263	330	220	220
407	405	361	395	306	363	225	226
Lip →	458	Lip →	466	367	408	290	286
551	550	575	576	404	432	295	310
551	550	656	655	429	475	365	367
613	633	Lop →	759	Lip →	478	375	387
Lop →	766	764	768	473	568	433	429
788	795			Lop →	764	447	447
				563	778	Lip →	465
				778		Lip →	484
						475	498
						490	557
						Lop →	773
						555	793
						790	
						799	

2.2 Optimized structures of 1:1 amine water complexes

The resulting optimized monohydrate structures of N555, MN4, MN5 and MMCN using B3LYP-D3/def2-TZVP on Orca 5.0.3 are provided in Tab. S4, S5, S6 and S7 for later application. The DFT predicted lower range wavenumber($<$ 800 cm^{-1}) amine modes are shown in the Tab. S3 to recognize six newly generated vibrations in monohydrates.

Table S4: xyz coordinates of N555 water structure in Å optimized at the B3LYP/TZ level on ORCA 5.0.3.

atom	x	y	z
N	0.054465648	-0.131388287	-1.164580209
C	-1.346800527	-0.104956655	-0.719872923
H	-1.834822501	0.738357484	-1.210411140
H	-1.829150253	-1.014290756	-1.082910163
C	0.729790115	1.106505794	-0.740894981
H	1.749062306	1.076110113	-1.127717406
H	0.226046864	1.941404585	-1.229418992
C	0.738682084	-1.292244504	-0.575912561
H	1.759545614	-1.305249575	-0.960089367
H	0.243212051	-2.192905263	-0.942813972
C	-1.446520032	0.004106448	0.827196224
H	-1.991178298	0.905010413	1.118377298
H	-1.990200425	-0.847453495	1.242567721
C	0.717715026	-1.232858889	0.976861413
H	1.733353528	-1.226191431	1.378718374
H	0.214351873	-2.108774415	1.392427824
C	0.712312337	1.257689737	0.805109011
H	1.728784825	1.308684861	1.201754721
H	0.206979337	2.180268484	1.099409822
C	-0.021284908	0.045821854	1.397167922
H	-0.054048215	0.120237893	2.485299860
O	0.370247049	0.033070667	-3.973002320
H	0.218378981	-0.093429781	-3.007839450
H	-0.053179872	-0.715547422	-4.402974192

Table S5: xyz coordinates of MN4 water structure in Å optimized at the B3LYP/TZ level on ORCA 5.0.3. The non-pairwise C-H of the methyl group is pointing away from the water unit.

atom	x	y	z
C	1.446088421	2.126430733	-4.805535734
C	2.069652861	3.251058211	-3.970089221
C	2.061429140	2.705233070	-2.516603521
C	1.435694405	1.310311759	-2.634614125
H	2.227310749	1.466599493	-5.219025837
H	1.461975502	4.151981159	-4.036242672
H	1.448456189	3.341290431	-1.880146765
H	3.072399118	3.495534206	-4.319170364
H	3.059463942	2.657260215	-2.082150298
H	0.819723336	1.036631447	-1.776872326
H	2.216113342	0.539719430	-2.752159906
H	0.837931104	2.488301740	-5.635775965
N	0.615843045	1.395258254	-3.845506212
C	0.127063147	0.118842212	-4.327673484
H	-0.483233112	0.267680469	-5.219896851
H	0.948200722	-0.571209319	-4.582330377
H	-0.492505679	-0.354211568	-3.564133865
O	-1.246361909	3.416471728	-3.097764420
H	-0.711403712	2.642030992	-3.385291009
H	-2.166041554	3.165533092	-3.223417210

Table S6: xyz coordinates of MN5 water structure in Å optimized at the B3LYP/TZ level on ORCA 5.0.3. The non-pairwise C-H of the methyl group is pointing away from the water unit.

atom	x	y	z
N	-0.765585687	-0.562105919	0.050867860
C	-1.899462452	-1.472336968	0.044446393
H	-1.589389789	-2.528488539	-0.008977614
H	-2.487476490	-1.335854998	0.953404609
H	-2.537484674	-1.261150556	-0.815179216
C	0.078086528	-0.764249469	1.232226490
H	0.492264484	-1.788788737	1.232096477
H	-0.554939380	-0.671811955	2.117230738
C	1.213487714	0.251606498	1.284238458
H	1.835295117	0.047555026	2.159259779
H	0.787594738	1.250214279	1.409806682
C	2.046501077	0.203064290	0.003921215
H	2.573973583	-0.756377326	-0.051499571
H	2.809883218	0.984024770	0.015354627
C	1.142810473	0.357348585	-1.218820639
H	1.713881535	0.229425838	-2.141571576
H	0.712703399	1.361901595	-1.235414026
C	0.009937484	-0.662162370	-1.188775059
H	-0.671551656	-0.497415986	-2.026006792
H	0.421315409	-1.682002932	-1.297936522
O	-1.528965853	2.182064158	0.192161339
H	-1.354897353	1.213896944	0.144024199
H	-2.485484212	2.279839231	0.210144088

Table S7: xyz coordinates of MMCN water structure in Å optimized at the B3LYP/TZ level on ORCA 5.0.3.

atom	x	y	z
C	0.125409372	-0.254881675	-1.121242219
C	0.340832207	1.038672936	-0.341188407
C	-0.204654596	0.939713820	1.084776980
C	0.392777999	-0.244553837	1.856942620
C	0.196064060	-1.539765800	1.057289250
C	0.739751400	-1.431456795	-0.368676414
H	0.556467125	-0.174108860	-2.122511884
H	-0.948645760	-0.426920963	-1.249479630
H	-0.134867837	1.879634607	-0.851643337
H	1.413417576	1.262713650	-0.305546786
H	0.004893747	1.876403380	1.601409934
H	-1.292039572	0.820460364	1.059818099
H	1.480035880	-0.069845943	1.967494702
H	0.691538445	-2.373838444	1.554370326
H	-0.873857834	-1.767735178	1.031436183
H	0.550853403	-2.368170128	-0.898759015
H	1.828457703	-1.307302632	-0.334332383
N	-0.203882648	-0.356438864	3.209294499
C	-0.060677338	0.868846695	3.992218063
H	-0.456387032	0.699092360	4.993561764
H	0.990972429	1.183912406	4.089267292
H	-0.626642275	1.682872006	3.547147139
C	0.326398869	-1.487085163	3.967498815
H	0.050644500	-2.430387167	3.503428680
H	1.423817943	-1.450610548	4.062531545
H	-0.099965130	-1.473915412	4.970596023
O	-2.937958259	-0.765687185	2.521305813
H	-3.479592081	-0.935225114	3.297613009
H	-2.019533128	-0.636941367	2.854430969

2.3 Torsional scans

To explore structural isomers and the softness of the free hydrogen torsional degrees of freedom, a suitable HO···NC torsion angle of the water against the amine was scanned for the N555, MN4, MN5 and MMCN monohydrates in steps of 5° over the full range, while all other coordinates were allowed to relax. The structure optimization was carried out using RIJCOSX-B3LYP-D3(BJ,abc)/ma-def2-TZVP[4,13] and RIJCOSX-B3LYP-D3(BJ,abc)/def2-TZVP[4,13]in Orca 5.0.3[7–10] with the keywords ABC SlowConv VeryTightOpt VeryTightSCF defgrid3 Mass2016. An example input file for relaxed surface scans is given in Tab. S10. To illustrate the sensitivity to computational detail, the relative energy of monohydrate structures of N555, MN4, MN5 and MMCN for relaxed surface scans are shown in Fig. S5 and S6 for two different basis sets. At selected torsional angles, the structure was reoptimized and the electronic, corrected zero point energy and the vibrational wavenumbers are calculated again using B3LYP-D3/ma-def2-TZVP[4,13] and B3LYP-D3/def2-TZVP[4,13] in Orca 5.0.3[7–10] with the keywords ABC defgrid3 VERYTIGHTSCF VERYTIGHTOPT FREQ. Some conformers like MN4+H_2O-b and MN4+H_2O-c are relaxed to the global minimum. To get a transition state, some conformers like MN4+H_2O-d are optimized using B3LYP-D3/ma-def2-TZVP[4,13] and B3LYP-D3/def2-TZVP[4,13]in Orca 5.0.3[7–10] with the keywords ABC UseSym SlowConv OptTS VeryTightOpt VeryTightSCF Freq defgrid3 Mass2016. The energy values and the lowest wavenumber(imaginary/real) are given in Tabs. S8 and S9.

Table S8: Relative electronic ΔE_{el} and zero point corrected ΔE_0 energy and lowest wavenumber $\tilde{\nu}$(imaginary/real) for different conformers using B3LYP-D3/ma-def2-TZVP. These conformers structures are shown in Fig. S1,S2,S3 and S4.

Conformer	ΔE_{el}/(kJ/mol)	ΔE_0/(kJ/mol)	lowest $\tilde{\nu}$/cm^{-1}
N555			
N555+H_2O-a	0.0000	0.0000	real
N555+H_2O-b	0.2186	-0.0019	imaginary
MN4			
MN4+H_2O-a	0.0000	0.0000	real
MN4+H_2O-d	1.7399	1.2239	imaginary
MN5			
MN5+H_2O-a	0.0000	0.0000	real
MN5+H_2O-c	1.7543	1.0057	imaginary
MN5+H_2O-d	1.7424	1.0662	real
MMCN			
MMCN+H_2O-a	0.0000	0.0000	real
MMCN+H_2O-d	0.2600	1.3312	imaginary

Table S9: Relative electronic ΔE_{el} and zero point corrected ΔE_0 energy and lowest wavenumber $\tilde{\nu}$(imaginary/real) for different conformers using B3LYP-D3/def2-TZVP. These conformers structures are shown in Fig. S1,S2,S3 and S4.

Conformer	ΔE_{el}/(kJ/mol)	ΔE_0/(kJ/mol)	lowest $\tilde{\nu}$/cm^{-1}
N555			
N555+H_2O-a	0.0000	0.0000	real
N555+H_2O-b	0.2598	-0.0148	imaginary
MN4			
MN4+H_2O-a	0.0000	0.0000	real
MN4+H_2O-d	1.4382	0.8995	imaginary
MN5			
MN5+H_2O-a	0.0000	0.0000	real
MN5+H_2O-c	0.9261	0.4874	imaginary
MN5+H_2O-d	0.9176	0.6693	real
MMCN			
MMCN+H_2O-a	0.0000	0.0000	real
MMCN+H_2O-b	0.0188	0.0317	real
MMCN+H_2O-d	1.1962	0.8335	imaginary

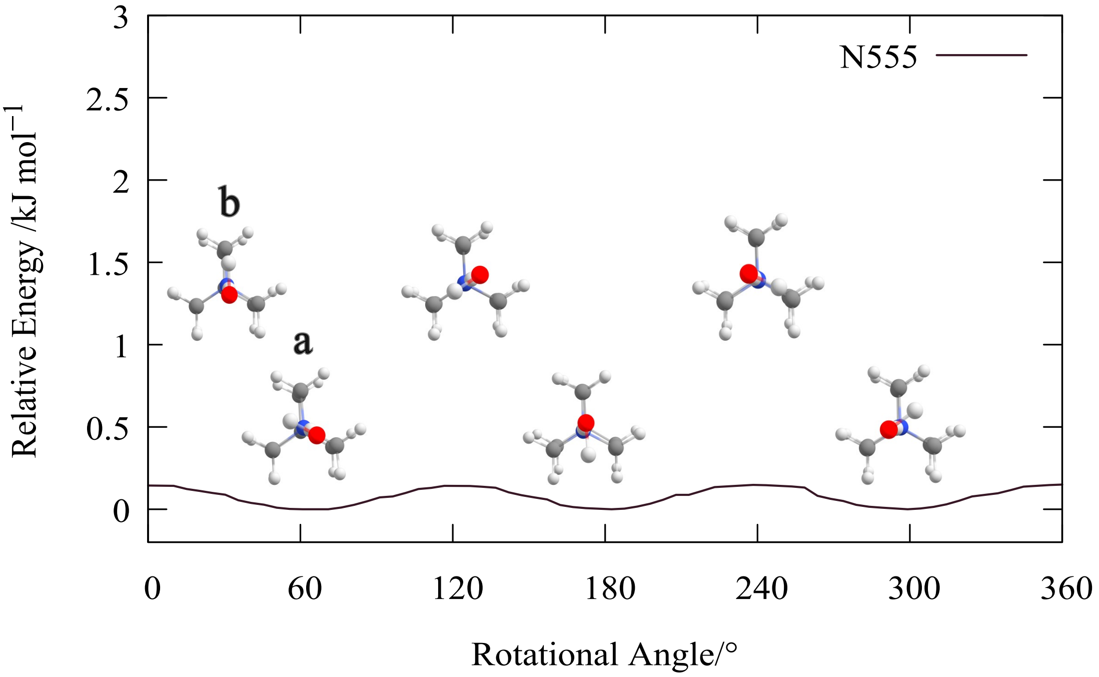

Figure S1: The torsional scan of N555+water with the basis set ma-def2-TZVP giving the relative energy of the torsion of the free OH bond of the water molecule over the amine rotating along the H-bond between water and nitrogen. As N555 is symmetric, three equivalent local minimum structures for N555 are found in the 360° torsional scan.

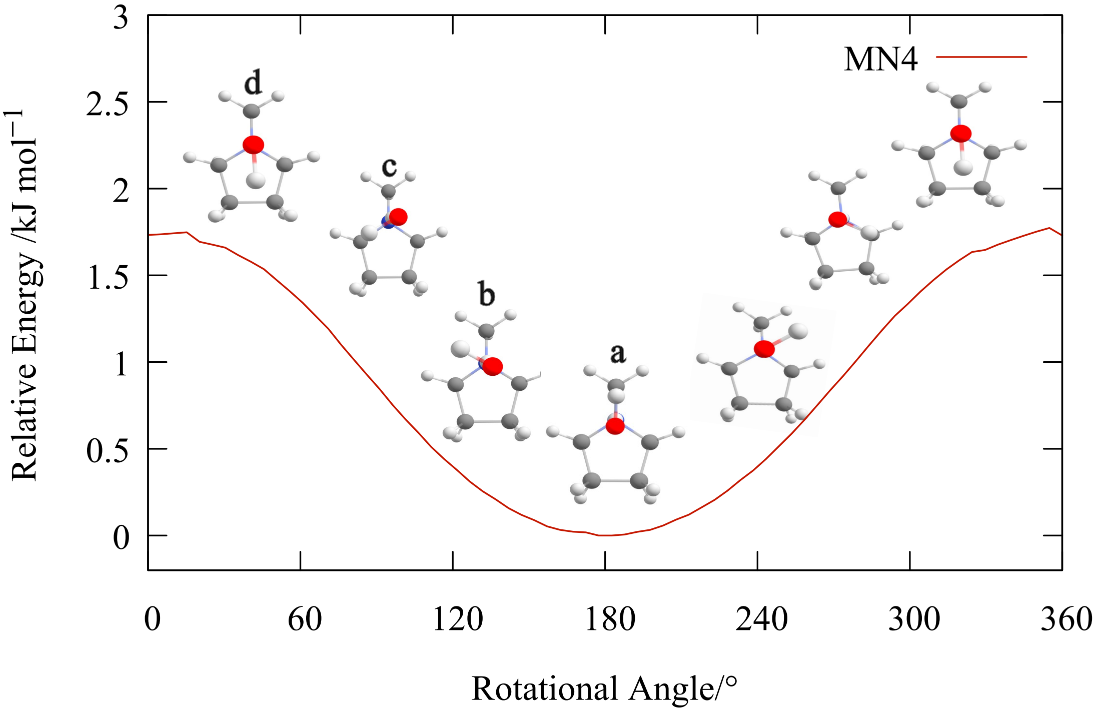

Figure S2: The torsional scan of MN4+water with the basis set ma-def2-TZVP giving the relative energy of the torsion of the free OH bond of the water molecule over the amine rotating along the H-bond between water and nitrogen. There is only one local minimum structure for MN4 monohydrate according to this scan.

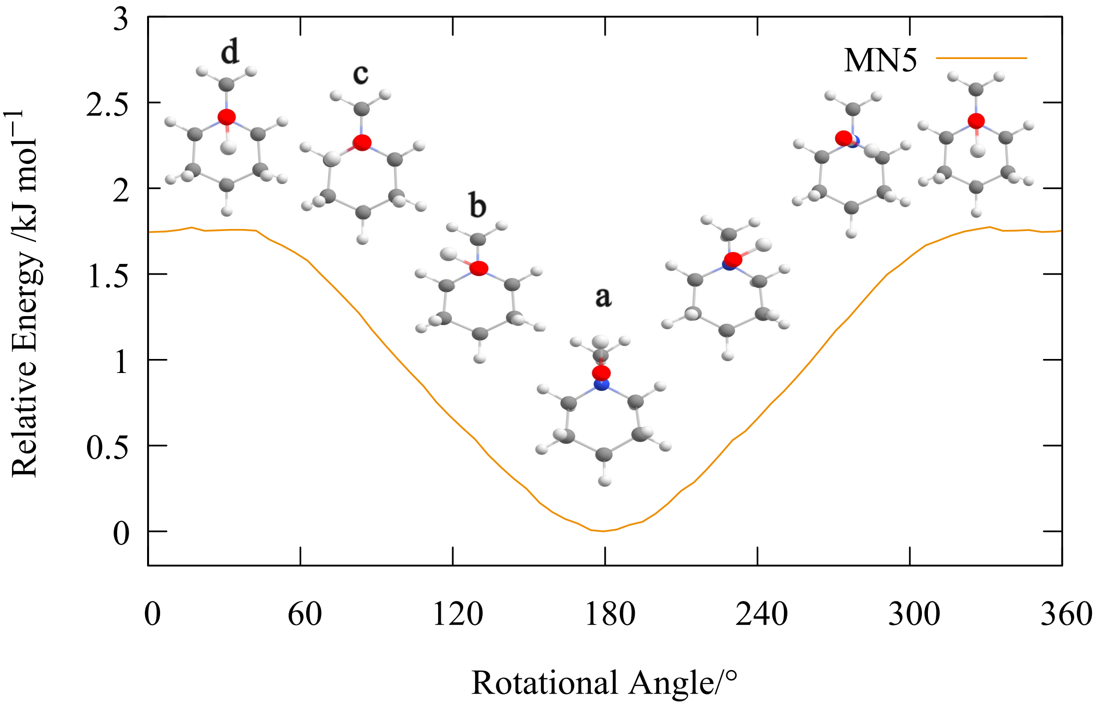

Figure S3: The torsional scan of MN5+water with the basis set ma-def2-TZVP giving the relative energy of the torsion of the free OH bond of the water molecule rotating along the H-bond between water and nitrogen over the amine. There is only one local minimum structure for MN5 monohydrate according to energy diagram.

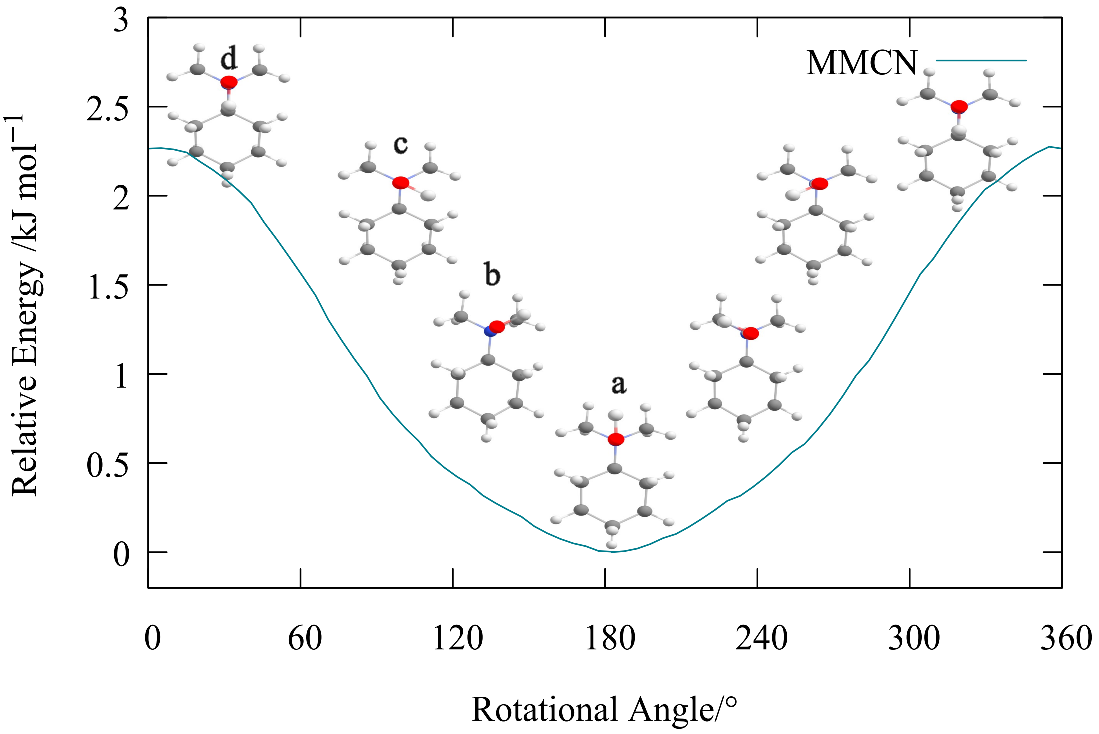

Figure S4: The torsional scan of MMCN+water with the basis set ma-def2-TZVP giving the relative energy of the torsion of the free OH bond of the water molecule rotating along the H-bond between water and nitrogen over the amine. There is only one local minimum structure for MMCN monohydrate according to energy diagram.

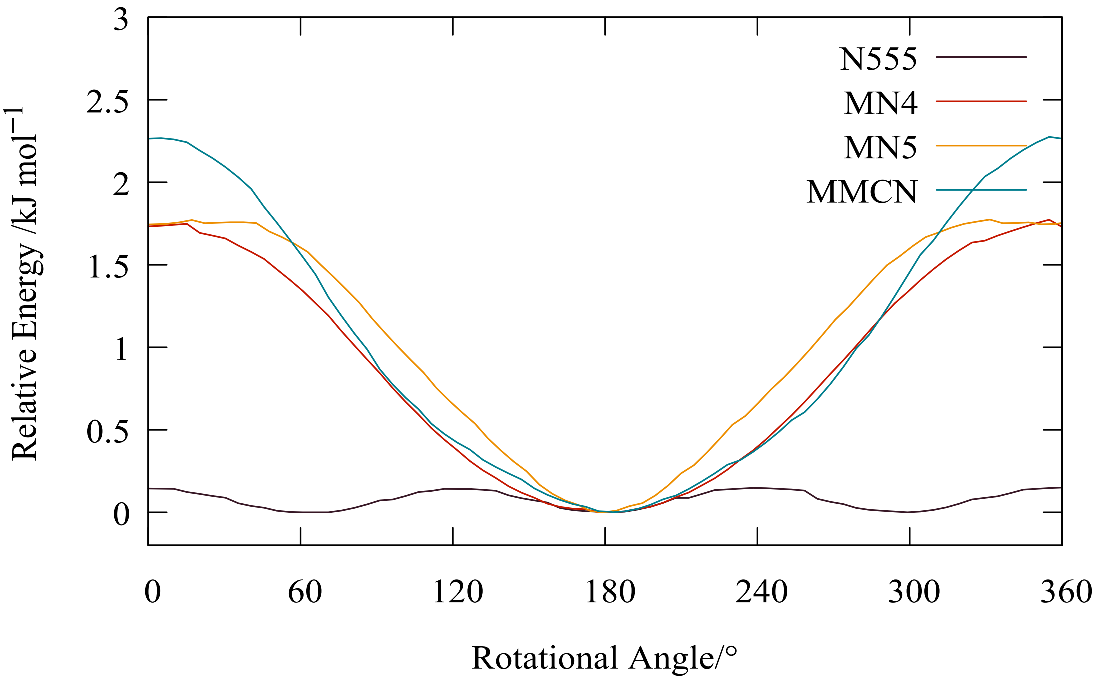

Figure S5: Comparison of the torsional scans for the monohydrates of N555, MN4, MN5 and MMCN using B3LYP-D3/ma-def2-TZVP

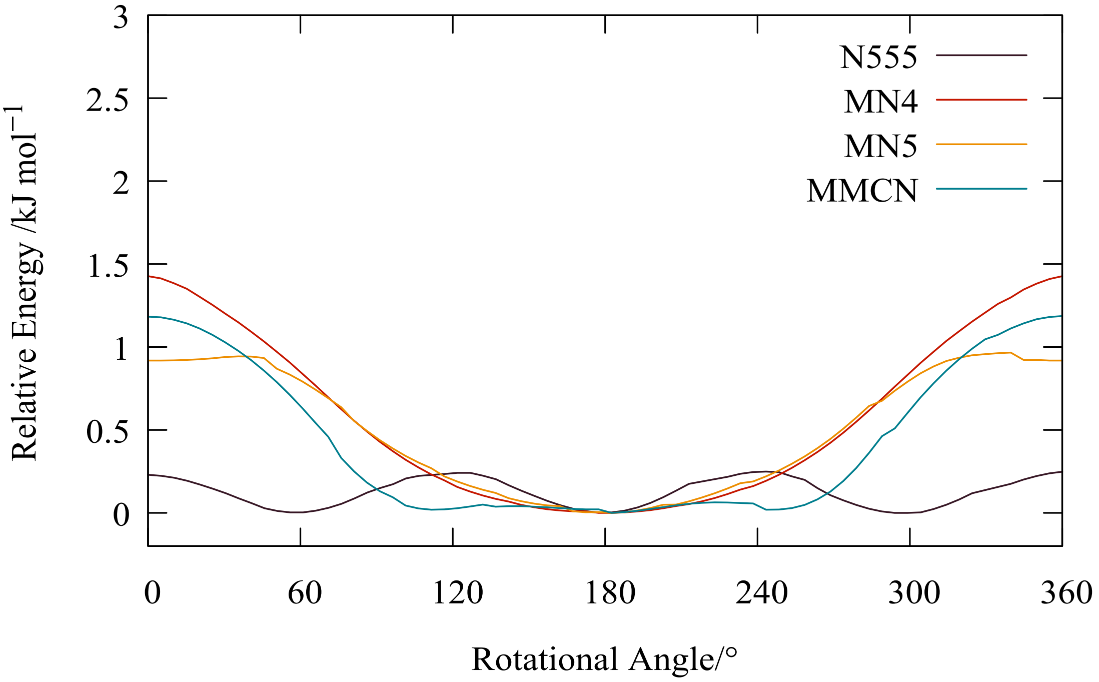

Figure S6: Comparison of the torsional scans for the monohydrates of N555, MN4, MN5 and MMCN using B3LYP-D3/def2-TZVP

2.4 Example input

For convenience, an example input for a relaxed coordinate scan is given in Tab. S10.

Table S10: Example input for relaxed surface scan in the ORCA 5.0.3 calculations at the B3LYP level of computation. In the input file, x x x x is needed to define the name of atoms HO...NC for torsion.

type of calculation	input
Relaxed surface scan	!B3LYP D3BJ ABC ma-def2-TZVP SlowConv VeryTightOpt<br>VeryTightSCF defgrid3 Mass2016<br>%geom Scan<br>D x x x x = 0, 360, 72 # Attention ORCA starts counting atom from 0<br>end<br>end<br>%pal nprocs 8 end<br>%Maxcore 3000<br>xyzfile 0 1 axstart.xyz

3 Integration methods

To calculate the coupling constants for the postulated resonance between OHb and b2 (Model A, described in the main text and next section) or the pairwise resonances between OHb, b2 and b2ON (Model B, described in the main text and next section), the experimental fractional intensities r of OHb, b2 and b2ON are required. The intensities in the range 3000 - 3380 cm^{-1} of the spectra shown in the Fig. 1 of the main text are evaluated. The fractional intensities are determined by four different integration methods and the mean values $\overline{r}$ as well as a measure for the scatter across the four methods Δs (*vide infra*) of these independent methods are taken as realistic estimates and uncertainties for the analysis of the resonance.
The four integration methods (I, II, III, IV) are described below and the resulting fractional intensities r and uncertainties Δr of each method are given in Tab S11. All four methods are largely based on the NoisySignalIntegration method by Nils O. B. Lüttschwager [14], which allows for different settings of the integration windows and their variation. In brief, the program analyzes the noise in a given wavenumber range of the spectrum and uses this to simulate additional noise with the same characteristics to many samples before the integration is performed. The uncertainty Δr is estimated as a 95% confidence interval of the resulting integral distributions and listed together with r in Tab. S11.

3.1 Method I

The more or less symmetric integration window around the band maximum is chosen so narrow that the signal remains consistently above the noise level. The integration boundaries are allowed to vary by 1 cm^{-1}. To give an example for the main band of the MN4 monohydrate, the low integration boundary is sampled in the 3285-3286 cm^{-1} range and the high boundary in the 3312-3313 cm^{-1} range.

3.2 Method II

The more or less symmetric integration window is larger, including significant noise traces beyond the wings of the signal, whose contributions should cancel on average for an ideal baseline. The integration boundaries are allowed to vary by 3 cm^{-1}. In the MN4 example, the boundaries are chosen from the ranges 3275-3278 cm^{-1} and 3320-3323 cm^{-1}.

3.3 Method III

The integration boundaries are allowed to vary between the extreme values of methods I and II. Staying with the MN4 example, the integration is carried out from 3275-3286 cm^{-1} to 3312-3323 cm^{-1}.

3.4 Method IV

The integration is based on a symmetric window between 30 and 40 cm^{-1} in size around the band maximum. For the main band of MN4, the maximum is at 3299cm^{-1} and the sampled integration window ranges from 3279-3284 cm^{-1} to 3314-3319 cm^{-1}.

3.5 Average intensity fraction

The fractional intensities from the four integration methods are averaged to obtain the estimated intensity fraction $\overline{r}$ listed in Tab. S11 for each band. To estimate the associated uncertainty due to the method used, the probability distribution histograms of all four integration methods are drawn together and the uncertainty Δs is calculated as one half of the span between the higher 1σ deviation of the highest histogram and the lower 1σ deviation of the lowest histogram. These results are depicted in Tab. S11.

4 Simple 3-state resonance models

4.1 Model A

In model A, there is only a 1:2 Fermi resonance between the bound OH stretching vibration of water (OHb) and the bending overtone of water (b2), explained in detail in the main text. The coupling constant W_2 is calculated using equation S1 with the ratio of the intensity fractions of OHb and b2 and the results are given in Tab. S12. The anharmonic peak position of OHb for Fig. 1 in the main text (dashed arrow) is derived from the equation S2.

Table S11: Experimental intensity ratio r and an error margin Δr of four integration methods and the average realistic intensity fraction $\overline{r}$ and uncertainty error Δs for b2, OHb and b2ON of amine-water complexes. The last column provides full widths at half maximum (FWHW) in cm^{-1} for the bands which are integrated.

	I		II		III		IV				FWHM
Code	r	Δr	r	Δr	r	Δr	r	Δr	$\overline{r}$	Δs	cm^{-1}
N555											
b2	0.30	0.01	0.28	0.02	0.29	0.01	0.29	0.01	0.29	0.03	4
OHb	0.51	0.02	0.50	0.01	0.51	0.02	0.52	0.02	0.51	0.04	6
b2ON	0.18	0.01	0.22	0.03	0.21	0.01	0.19	0.01	0.20	0.05	4
MN4											
b2	0.21	0.02	0.23	0.04	0.23	0.02	0.19	0.02	0.21	0.07	8
OHb	0.62	0.02	0.59	0.03	0.62	0.02	0.66	0.03	0.61	0.07	8
b2ON	0.17	0.01	0.18	0.03	0.15	0.02	0.15	0.02	0.18	0.05	8
MN5											
b2	0.23	0.01	0.25	0.03	0.25	0.02	0.21	0.01	0.23	0.06	8
OHb	0.64	0.02	0.60	0.03	0.61	0.01	0.64	0.02	0.63	0.07	7
b2ON	0.13	0.02	0.15	0.01	0.14	0.01	0.15	0.01	0.14	0.04	5
MMCN											
b2	0.28	0.01	0.28	0.01	0.28	0.01	0.28	0.01	0.28	0.03	6
OHb	0.52	0.02	0.52	0.02	0.52	0.01	0.53	0.01	0.52	0.05	8
b2ON	0.20	0.01	0.20	0.01	0.20	0.01	0.19	0.01	0.20	0.02	8

$$W_2 = \Delta\tilde{\nu}_{\mathrm{b2,OHb}} \sqrt{\frac{\frac{\overline{r}_{\mathrm{OHb}}}{\overline{r}_{\mathrm{b2}}}}{(\frac{\overline{r}_{\mathrm{OHb}}}{\overline{r}_{\mathrm{b2}}}+1)^2}} \tag{S1}$$

$$\tilde{\nu}_{\mathrm{A}} = \frac{\overline{r}_{\mathrm{b2}} \cdot \tilde{\nu}_{\mathrm{b2}} + \overline{r}_{\mathrm{OHb}} \cdot \tilde{\nu}_{\mathrm{OHb}}}{\overline{r}_{\mathrm{b2}} + \overline{r}_{\mathrm{OHb}}} \tag{S2}$$

4.2 Model B

The resonances (2+1):1 and (2:1) are considered sequentially as shown in the right part of the Fig. 3 and further described in the main text. The coupling constant W_3 and the deperturbed $\tilde{\nu}(\mathrm{OHb}^0)$ between OHb and b2ON are calculated by using equation S4 and S3. To examine the coupling constant W_2 between the b2ON-deperturbed OHb^0 and b2, the equation S5 is used in model B. The fully deperturbed position of OHb in model B is determined with equation S6 and is shown in Fig. 1 of the main text (solid arrow). All results are shown in Tab. S12.

$$\tilde{\nu}(\mathrm{OHb}^0) = \frac{\overline{r}_{\mathrm{OHb}} \cdot \tilde{\nu}(\mathrm{OHb}^{\mathrm{exp}}) + \overline{r}_{\mathrm{b2ON}} \cdot \tilde{\nu}(\mathrm{b2ON}^{\mathrm{exp}})}{\overline{r}_{\mathrm{OHb}} + \overline{r}_{\mathrm{b2ON}}} \tag{S3}$$

$$W_3 = \Delta\tilde{\nu}_{\mathrm{b2ON,OHb}} \sqrt{\frac{\frac{\overline{r}_{\mathrm{OHb}}}{\overline{r}_{\mathrm{b2ON}}}}{(\frac{\overline{r}_{\mathrm{OHb}}}{\overline{r}_{\mathrm{b2ON}}}+1)^2}} \tag{S4}$$

$$W_2 = \Delta\tilde{\nu}_{\mathrm{b2,OHb^0}} \sqrt{\frac{\frac{(\overline{r}_{\mathrm{b2ON}}+\overline{r}_{\mathrm{OHb}})}{\overline{r}_{\mathrm{b2}}}}{(\frac{(\overline{r}_{\mathrm{b2ON}}+\overline{r}_{\mathrm{OHb}})}{\overline{r}_{\mathrm{b2}}}+1)^2}} \tag{S5}$$

$$\tilde{\nu}_{\mathrm{B}} = \frac{\overline{r}_{\mathrm{b2}} \cdot \tilde{\nu}_{\mathrm{b2}} + \overline{r}_{\mathrm{OHb}} \cdot \tilde{\nu}_{\mathrm{OHb}} + \overline{r}_{\mathrm{b2ON}} \cdot \tilde{\nu}_{\mathrm{b2ON}}}{\overline{r}_{\mathrm{b2}} + \overline{r}_{\mathrm{OHb}} + \overline{r}_{\mathrm{b2ON}}} \tag{S6}$$

Table S12: Experimental peak position $\tilde{\nu}$, (2:1)-deperturbed peak position $\tilde{\nu}_A$, ((2+1):1)-deperturbed peak position $\tilde{\nu}_B$, interim b2ON-deperturbed peak position $\tilde{\nu}(\mathrm{OH}^0)$ and the coupling constants for resonance of OHb according to models A and B.

		A		B			
Code	$\tilde{\nu}$	$\tilde{\nu}_A$	W_2	$\tilde{\nu}_B$	$\tilde{\nu}(\mathrm{OH}^0)$	W_2	W_3
N555	3298	3260	51	3278	3313	55	24
MN4	3299	3271	48	3284	3310	49	21
MN5	3289	3262	45	3274	3299	47	21
MMCN	3282	3249	46	3268	3299	51	28

4.3 Exemplary sensitivity analysis

To illustrate the sensitivity of the coupling parameters and deperturbed band positions to the experimentally derived intensities, one can explore different combinations of intensities within the experimental uncertainty. For N555 the following extreme (normalized) intensity values are assumed instead of the average (0.29,0.51,0.20) intensity triple: high coupling limit (0.31,0.45,0.24), low coupling limit (0.27,0.57,0.16), high wavenumber scenario (0.26,0.49,0.25) and low wavenumber scenario (0.32,0.53,0.15). The coupling constants and anharmonic wavenumbers of model A and B for these cases are given in Tab. S13. Rewardingly, the coupling constants change by less than $\pm 3\,\mathrm{cm}^{-1}$ and the deperturbed band positions by less than $\pm 6\,\mathrm{cm}^{-1}$.

Table S13: Experimental peak position $\tilde{\nu}$, (2:1)-deperturbed peak position $\tilde{\nu}_A$, ((2+1):1)-deperturbed peak position $\tilde{\nu}_B$, interim b2ON-deperturbed peak position $\tilde{\nu}(\mathrm{OH}^0)$ and the coupling constants of OHb for extreme intensity values according to models A and B.

					A		B			
Case	$\overline{r}_{\mathrm{b2}}$	$\overline{r}_{\mathrm{OHb}}$	$\overline{r}_{\mathrm{b2ON}}$	$\tilde{\nu}$	$\tilde{\nu}_A$	W_2	$\tilde{\nu}_B$	$\tilde{\nu}(\mathrm{OH}^0)$	W_2	W_3
Average	0.29	0.51	0.20	3298	3260	51	3278	3313	55	24
High coupling	0.31	0.45	0.24	3298	3255	52	3278	3316	57	25
Low coupling	0.27	0.57	0.16	3298	3264	49	3278	3310	52	22
High wavenumber	0.26	0.49	0.25	3298	3262	50	3284	3316	54	25
Low wavenumber	0.32	0.53	0.15	3298	3258	51	3272	3310	54	22

5 Experimental wavenumbers and scaled harmonic predictions

Comparisons between theory and experiment are given in Tab. S14.

Table S14: Experimentally observed wavenumbers $\tilde{\nu}$ compared to harmonic B3LYP/TZ predictions ω unscaled and scaled by 0.97 for the hydrates of N555, MN4, MN5 and MMCN for figure 1, 4 and 5 in the main document. The positions of the approximate band maxima in the warm spectrum of MN4, which is depicted in figure 5, are given in parenthesess. The signals that cannot be securely assigned are listed as b2±X where X could be t (torsion), ip (in plane), or op (out of plane) motion.

Species	$\tilde{\nu}$/cm^{-1}	ω/cm^{-1}	0.97 ω/cm^{-1}
N555			-
b2	3193	3318	-
OHb	3298	3366	3265
b2ON	3351	3484	-
C	3441	-	-
T	3475	3510	-
MN4			-
b2	3190 (≈3198)	3312	-
b2−X	(≈3153)	-	-
b2+X	(≈3228)	-	-
OHb	3299 (≈3312)	3384	3282.5
b2ON	3348 (≈3352)	3474	-
TAWbA	3404	3506	-
TAWbA	3455	3565	-
C	3445	-	-
TWbWA	3485	3532	-
TWfWfA	3722	3852	-
OHf	3712	3848	-
MN5			-
b2	3188	3318	-
OHb	3289	3369	3267.9
b2ON	3343	3471	-
C	3430	-	-
T	3482	3531	-
MMCN			-
b2	3186	3322	-
OHb	3282	3360	3259.2
b2ON	3346	3487	-
C	3428	-	-
T	3479	3518	-

References

[1] H. C. Gottschalk, T. L. Fischer, V. Meyer, R. Hildebrandt, U. Schmitt, M. A. Suhm, *Instruments* **2021**, *5*, 12.
[2] S. Grimme, J. Antony, S. Ehrlich, H. Krieg, *J. Chem. Phys.* **2010**, *132*, 154104.
[3] S. Grimme, S. Ehrlich, L. Goerigk, *J. Comput. Chem.* **2011**, *32*, 1456–1465.
[4] F. Weigend, R. Ahlrichs, *Phys. Chem. Chem. Phys.* **2005**, *7*, 3297–3305.
[5] W. Sander, S. Roy, I. Polyak, J. M. Ramirez-Anguita, E. Sanchez-Garcia, *J. Am. Chem. Soc.* **2012**, *134*, 8222–8230.
[6] D. Leicht, M. Kaufmann, R. Schwan, J. Schäfer, G. Schwaab, M. Havenith, *J. Chem. Phys.* **2016**, *145*, 204305.
[7] F. Neese, *Wiley Interdiscip. Rev. Comput. Mol. Sci.* **2012**, *2*, 73–78.
[8] F. Neese, *Wiley Interdiscip. Rev. Comput. Mol. Sci.* **2022**, *12*, e1606.
[9] M. A. Marques, M. J. Oliveira, T. Burnus, *Computer Physics Communications* **2012**, *183*, 2272–2281.
[10] S. Lehtola, C. Steigemann, M. J. Oliveira, M. A. Marques, *SoftwareX* **2018**, *7*, 1–5.
[11] S. Grimme, *J. Chem. Theory Comput.* **2019**, *15*, 2847–2862.
[12] P. Pracht, F. Bohle, S. Grimme, *Phys. Chem. Chem. Phys.* **2020**, *22*, 7169–7192.
[13] F. Weigend, *Phys. Chem. Chem. Phys.* **2006**, *8*, 1057–1065.
[14] N. O. B. Lüttschwager, *Journal of Open Source Software* **2021**, *6*, 3526.
