# Supplementary material for: Microhydration of Tertiary Amines: Robust Resonances in Red-Shifted Water
Source: J Phys Chem Lett. 2023 Nov 6;14(45):10194–9. doi: 10.1021/acs.jpclett.3c02517 (PMC10658632; doi:10.1021/acs.jpclett.3c02517)
Supplement: Supplementary file 2 — jz3c02517_si_002.pdf [file jz3c02517_si_002.pdf]

jz-2023-02517s.R1

Name: Peer Review Information for "Microhydration of Tertiary Amines: Robust Resonances in Red-Shifted Water"

First Round of Reviewer Comments

Reviewer: 1

Comments to the Author

Jz-2023-02517s
Title: Microhydration of Tertiary Amines: Robust Resonances in Red-Shifted Water

AUTHORS: Lwin, Eaindra; Fischer, Taija Lena; Suhm, Martin

This manuscript describes a joint experimental and computational study of the infrared spectroscopy of a series of tertiary amines, focusing attention on the 1:1 complex with water under the cold conditions of a slit supersonic expansion. The quality of the spectra is high, and the analysis is carried out with care. The authors first establish that a triad of transitions dominates the IR spectrum in the region of the Hbonded OH stretch fundamental of the complexes, where only a single transition should exist. They then consider various possibilities, leading to assignment as a Fermi triad in which the OH bend overtone and a combination band of the O⋯N stretch with the bend overtone anharmonically mix with the OH stretch, gaining intensity from the OH stretch.

It appears that previous studies using IR-VUV and matrix isolation misassigned which transitions are due to the 1:1 complex, and therefore have made deductions regarding the vibrational dynamics that were incorrect.

I am struggling to decide whether this manuscript belongs in JPC Letters. The earlier IR-VUV study was in JPCL, so in that regard, correcting that work in the same journal certainly makes sense. But the 'hook' that attracts the reader to this manuscript is hard to find as now written and connecting to vibrational dynamics isn't particularly persuasive once found. Furthermore, the main text is a bit of a slog to read without significant modification.

(1) I suggest that a better entry into the subject might be to have a figure that illustrates limiting cases of what happens to a H-bonded OH stretch fundamental as a function of the strength of the H-bond – showing increasing shifts, then the turn-on of FR with the overtone of the bend, raise the possibility that there can be Franck-Condon like transitions of a low-frequency intermolecular vibration built off the H-bonded OH stretch. Then they could set up their case as a hybrid in which the Franck-Condon like low-frequency combination band is built off the dark state (the overtone of the bend) rather than directly off the H-bonded OH stretch fundamental. Is this something that hasn't been considered previously?

(2) The reader is taken through the various possibilities for how to assign the three bands before addressing what many readers would be concerned about at the outset; namely, that one or more of the bands was due to a different sized cluster. They carefully cross off this possibility by changing the concentrations of water and amine in the expansion, as shown in Figure 4. Here the stability of the expansion enables them to take difference spectra that reveal bands whose intensity changes differently than the triad that is present at lowest concentrations of water and amine. This reviewer would have appreciated such arguments to come earlier in the manuscript, so they didn't seem like an after-thought.

(3) The figures have legends at the top that are difficult to decipher for the reader. I found myself spending more time than necessary trying to sort through the color scheme, acronyms, concentration, and backing pressure conditions.

(4) Table 1 could probably go in supplementary information. Wouldn't it be more persuasive if predicted frequencies and intensities were compared against experiment?

Reviewer: 2

Comments to the Author

This letter reports infrared (IR) spectra of the binary complexes of tertiary amine and water in the OH stretch region. The systematic study demonstrates the robust Fermi resonance between hydrogen-bonded OH stretch and its bending overtone (and further coupling with a combination band might exist). This study is well designed to make use of the unique advantage of FTIR spectroscopy in pulsed slit jet expansion. The concentration/pressure dependence of the spectra seems to strongly support the size assignments of the observed bands, suggesting contamination of higher clusters in the previously reported spectra of the related studies. This study offers the benchmark data for precise analyses of the Fermi resonance in hydrogen-bonded neutral water, and their importance is quite high. This letter would attract great interest of the broad community. I recommend publication of this manuscript in J. Phys. Chem. Lett. Though this is a well-written manuscript, I request the authors to examine some minor points prior to publication.

1. In the spectrum of N555, the band shape of OHb is clearly asymmetric and a shoulder is seen at the low frequency side. What is the origin of this shoulder? Is this shoulder included in the estimation of the intensity of the OHb band?

2. It might be helpful for readers if the information on the bandwidth is also summarized in SI.

3. I do not understand merit of the use of the original codes (e.g., N555) for the tertiary amine samples. In the present systems the Fermi resonance seems robust and no clear structure dependence is found. Moreover, impact of on the amine structures is not discussed. Therefore, meaning of the code based on the amine structure is unclear. I agree that use of the chemical names is also tedious, and this comment is not strong objection.

Reviewer: 3

Comments to the Author

The paper documents the spectral signatures of an important class of H-bonds, those in hydrates of a strong acceptor molecular family, namely amines. Using an experimental comparative careful approach (using absorption measurements in a free jet set-up), the authors document a spectral triad in the 3100-3400 cm-1 region as a characteristic feature of the water OH stretching engaged in a strong H-bond together with its coupling to the water bending overtone (Fermi resonance) and another mode. Thanks to general considerations, a careful concentration study and quantum chemistry calculations, the nature of this latter mode is discussed and eventually assigned to the H-bond O···N stretch. The paper delivers here a convincing assignment of the spectral features of a water-amine system, which enlightens previous recent works on a comparable system (water-trimethylamine) since it leads us to totally reconsider these previous assignments.

The paper is well written and conveys an important message for the molecular spectroscopy community. In my opinion, it deserves to be published in JPCL, providing that the few points listed below are documented.

- Figure 1 caption : The last sentence (arrows' meaning and intensity weighted …) appears weird and obscure when one reads the caption for the first time (when cited in the text). I suggest to label the arrows as unperturbed positions and refer to the text for details of the deperturbation models

- Trimethylamine is a natural model of the water···N H-bonds targeted. Is there any reason why it is not included in the present study ? Inasmuch as the two studies evoked in the discussion (and whose assignment is clearly questioned) reported on this very system.

- One of the merits of the paper is the reassignment of these previous works. I would have expected more extended developments, in particular :

= for the matrix study, the fact that with trimethylamine a triad is satisfactorily observed at nearly the same locations as in the MMCN molecule.

= for the IR/VUV experiment the absence of a band assignable to a OHb feature and of a triadic pattern as well, is a strong hint at the occurrence of extensive fragmentation processes in the ion, which ruin the claimed mass-selective character of the experiment.

Author's Response to Peer Review Comments:

We thank the three reviewers for their careful reading, literature coverage and commenting of our manuscript, including valuable advice for improvement. We reproduce their comments (in red) and provide answers (in black), highlighting the resulting text changes (in blue).

Reviewer: 1

Recommendation: This paper is probably publishable, but major revision is needed; I do not need to see future revisions.

This manuscript describes a joint experimental and computational study of the infrared spectroscopy of a series of tertiary amines, focusing attention on the 1:1 complex with water under the cold conditions of a slit supersonic expansion. The quality of the spectra is high, and the analysis is carried out with care. The authors first establish that a triad of transitions dominates the IR spectrum in the region of the Hbonded OH stretch fundamental of the complexes, where only a single transition should exist. They then consider various possibilities, leading to assignment as a Fermi triad in which the OH bend overtone and a combination band of the O…N stretch with the bend overtone anharmonically mix with the OH stretch, gaining intensity from the OH stretch.

It appears that previous studies using IR-VUV and matrix isolation misassigned which transitions are due to the 1:1 complex, and therefore have made deductions regarding the vibrational dynamics that were incorrect. I am struggling to decide whether this manuscript belongs in JPC Letters. The earlier IR-VUV study was in JPCL, so in that regard, correcting that work in the same journal certainly makes sense. But the 'hook' that attracts the reader to this manuscript is hard to find as now written and connecting to vibrational dynamics isn't particularly persuasive once found. Furthermore, the main text is a bit of a slog to read without significant modification.

We initially also struggled somewhat whether JPC Letters would be the correct format for our work, but found it adequate for two reasons:

1. Letters are (also in their historical use) iterative approaches to the truth and letter writers can mutually assist this approach to the true picture. We have deliberately not directly studied the system whose interpretation we are ultimately challenging (this is work which has started in the last few days in our and other labs and will likely result in a systematic and well-secured study of the parent complex trimethylamine-water), but rather we have first explored an apparently repeating spectroscopic pattern for a number of amines, also giving the triggering work the chance to critically reconsider their spectral interpretation (as should ideally already have been the case for the water dimer controversy, also published in JPC Letters by both sides). Our observed resonance pattern is surprisingly new, general and simple and it will for sure induce multiple activity on the modeling side to be fully grasped.

2. The second reason is that Letters are supposed to be short preliminary reports, ideally to be followed up by something much more extensive. Indeed, we will expand the chemical and spectroscopic space in a large follow-up contribution, so in retrospective this Letter will likely be viewed as an initial trigger of a new subfield of vibrational resonance in strong neutral hydrogen bonds.

Suggestions to sharpen these messages are very welcome, see below.

(1) I suggest that a better entry into the subject might be to have a figure that illustrates limiting cases of what happens to a H-bonded OH stretch fundamental as a function of the strength of the H-bond – showing increasing shifts, then the turn-on of FR with the overtone of the bend, raise the possibility that there can be Franck-Condon like transitions of a low-frequency intermolecular

vibration built off the H-bonded OH stretch. Then they could set up their case as a hybrid in which the Franck-Condon like low-frequency combination band is built off the dark state (the overtone of the bend) rather than directly off the H-bonded OH stretch fundamental. Is this something that hasn't been considered previously?

We take up this useful suggestion of summarizing our interpretation of the spectra in a concise picture but think that this may be better placed at the end of the manuscript, summing up the collected evidence we provide. However, we now insert a guiding sentence in this direction in the introductory paragraph:

…interferes with the dominant intramolecular resonance. Surprisingly, this does not happen in the form of familiar Franck-Condon like progressions on top of the hydrogen bonded OH stretch, but instead on top of the initially dark bend overtone resonance partner, perhaps through more indirect [https://doi.org/10.1021/acs.jpca.1c04264] coupling mechanisms. These infrared spectroscopic signatures of anharmonic motion…

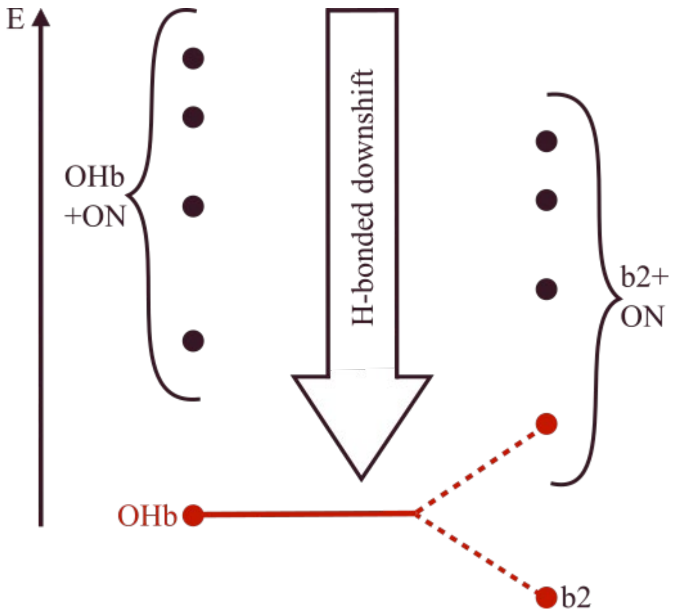

The picture itself is introduced as Fig. 6 with the caption:

In monohydrates of tertiary amines, the hydrogen-bonded and thus downshifted OHb stretching mode does not light up intermolecular ON combination bands on top of OHb (left) but rather on top of the Fermi-resonant bending overtone b2 (right, red dashes symbolizing redistribution of OHb infrared intensity).

It is inserted into the summarizing text as:

…third systematic spectral feature that likely involves a combined inter-intramolecular excitation, which surprisingly builds on the dark intramolecular state (Fig. 6) and that contributes…

(2) The reader is taken through the various possibilities for how to assign the three bands before addressing what many readers would be concerned about at the outset; namely, that one or more of the bands was due to a different sized cluster. They carefully cross off this possibility by changing the concentrations of water and amine in the expansion, as shown in Figure 4. Here the stability of the expansion enables them to take difference spectra that reveal bands whose intensity changes differently than the triad that is present at lowest concentrations of water and amine. This reviewer would have appreciated such arguments to come earlier in the manuscript, so they didn't seem like an after-thought.

Again, we now better assist the flow of arguments by ruling out contributions of other cluster sizes at an earlier stage:

…dilution of the amine and water to obtain rotationally and vibrationally cold dimers, essentially free of contributions from larger clusters. For comparison…

(3) The figures have legends at the top that are difficult to decipher for the reader. I found myself spending more time than necessary trying to sort through the color scheme, acronyms, concentration, and backing pressure conditions.

We agree that the legends sometimes have a more documentary than initially guiding role and we improve the situation by a few measures, without losing the documentary information for readers who want to spend more time on these details.

In Fig. 1, we put the non-essential varying information in parenthesis in the style of N555 (at 0.1 hPa + H_2O at 0.4 hPa) and we complement each chemical formula with its acronym directly below it, in the color of the figure trace.

In Fig. 4, we do the same in the style of MN4 (at 2.0 hPa) H_2O (at 2.0 hPa) He (at 750 hPa), and in addition we label each spectral trace in its color by an appropriate qualitative descriptor:

high pressure, more water / high pressure, less water / difference / low pressure, more water / difference / low pressure, less water

In Fig. 5, we also organize the legend with parentheses and now use low pressure - high concentration and high pressure - low concentration as colored descriptive trace labels.

(4) Table 1 could probably go in supplementary information. Wouldn't it be more persuasive if predicted frequencies and intensities were compared against experiment?

We agree that Table 1 could be shortened (e.g. by only providing values for MN4 in the main text and the three others in the supplementary information), but part of our attempted persuasiveness is to show that four different amines show essentially the same coupling situation. In our field, such anharmonic couplings are often viewed as coincidental happenings for specific compounds, whereas our key JPC Letters message is one of *systematic* patterns across different compounds, as one finds them for group frequencies. Understand one, understand all of them.

The reviewer proposal to replace the table by one which compares frequencies and intensities between theory and experiment in our opinion does not work because harmonic theory (last column) does not explain the intensity pattern at all and the coupling models A and B are designed to exactly reproduce the experimental intensity and frequency pattern for two or three peaks, so there is no comparison possible. The comparison happens via the coupling constants which are needed to *reproduce* experimental intensities. Of course, we more than welcome and expect future theory work to make forward predictions of frequencies and intensities, but currently we can only provide backward analysis of the experimental frequencies and intensities in terms of effective coupling constants (whose similarity explains the similar situation in all compounds).

Reviewer: 2

Recommendation: This paper is publishable subject to minor revisions noted. Further review is not needed.

Comments:
This letter reports infrared (IR) spectra of the binary complexes of tertiary amine and water in the OH stretch region. The systematic study demonstrates the robust Fermi resonance between hydrogenbonded OH stretch and its bending overtone (and further coupling with a combination band might exist). This study is well designed to make use of the unique advantage of FTIR spectroscopy in pulsed slit jet expansion. The concentration/pressure dependence of the spectra seems to strongly support the size assignments of the observed bands, suggesting contamination of higher clusters in the previously reported spectra of the related studies. This study offers the benchmark data for precise analyses of the Fermi resonance in hydrogen-bonded neutral water, and their importance is quite high. This letter would attract great interest of the broad community. I recommend publication of this manuscript in J. Phys. Chem. Lett. Though this is a well-written manuscript, I request the authors to examine some minor points prior to publication.

1. In the spectrum of N555, the band shape of OHb is clearly asymmetric and a shoulder is seen at the low frequency side. What is the origin of this shoulder? Is this shoulder included in the estimation of the intensity of the OHb band?

This is a very accurate observation, the discussion of which we planned to shift to a full paper because we have no unambiguous answer, yet. All four systems show some asymmetries on either side of the main peak but N555 shows the strongest shoulder. We decided to include it into the integration, because it is possible that in the other systems an analogous contribution is just hidden, but we cannot rigorously exclude at this stage that it is due to a trimer. Based on the spectra at different concentrations we have,

it is somewhat more likely that it belongs to the dimer, possibly due to some secondary resonance. This will be addressed in more detail in a full paper, assisted by 18O isotope substitution and concentration series.

We now write:

…a strong central band is flanked by two more narrow and weaker satellites. The central band has some fine structure, most prominently for N555, which we assume to be due to subtle resonances, but contributions from larger clusters can not always be strictly ruled out. The lower frequency…

…the integrated intensity of the observed transitions (including any fine structure on the wings), obtained by different…

2. It might be helpful for readers if the information on the bandwidth is also summarized in SI.

We now include into Table S11 in the SI a column with the FWHM of all 12 bands employed in the resonance analysis.

3. I do not understand merit of the use of the original codes (e.g., N555) for the tertiary amine samples. In the present systems the Fermi resonance seems robust and no clear structure dependence is found. Moreover, impact of on the amine structures is not discussed. Therefore, meaning of the code based on the amine structure is unclear. I agree that use of the chemical names is also tedious, and this comment is not strong objection.

Indeed, for the four amines and their monotonous behaviour, a range of different codings including the chemical names would be possible. With the long-term goal of the HyDRA benchmark on the predictability of OH stretching wavenumbers in mind (original Ref. (20)), we have chosen a code which can be systematically extended to our target of more than 10 different amine monohydrates.

Reviewer 3:

Recommendation: This paper is publishable subject to minor revisions noted. Further review is not needed.

Comments:
The paper documents the spectral signatures of an important class of H-bonds, those in hydrates of a strong acceptor molecular family, namely amines. Using an experimental comparative careful approach (using absorption measurements in a free jet set-up), the authors document a spectral triad in the 3100-3400 cm-1 region as a characteristic feature of the water OH stretching engaged in a strong H-bond together with its coupling to the water bending overtone (Fermi resonance) and another mode. Thanks to general considerations, a careful concentration study and quantum chemistry calculations, the nature

of this latter mode is discussed and eventually assigned to the Hbond O···N stretch. The paper delivers here a convincing assignment of the spectral features of a water-amine system, which enlightens previous recent works on a comparable system (watertrimethylamine) since it leads us to totally reconsider these previous assignments.
The paper is well written and conveys an important message for the molecular spectroscopy community. In my opinion, it deserves to be published in JPCL, providing that the few points listed below are documented.

- Figure 1 caption : The last sentence (arrows' meaning and intensity weighted …) appears weird and obscure when one reads the caption for the first time (when cited in the text). I suggest to label the arrows as unperturbed positions and refer to the text for details of the deperturbation models

We agree that one should be focused more on the initial viewing of this figure and we follow the reviewer suggestion:

In the figure itself, we mark the arrows as deperturbed OHb positions. In the caption, we replace:

…, as are the intensity-weighted average positions of OHb and b2 (dashed) and OHb, b2 and b2ON (continuous arrows).

by

. Dashed and continuous arrows refer to deperturbed OHb positions, based on intensities and positions of two or three experimental signals, respectively. See text for further details.

- Trimethylamine is a natural model of the water···N H-bonds targeted. Is there any reason why it is not included in the present study ? Inasmuch as the two studies evoked in the discussion (and whose assignment is clearly questioned) reported on this very system.

As a matter of fact, we are experimentally studying the trimethylamine monohydrate only since a couple of weeks, and we plan an extensive characterization involving isotope substitution and multiple experiments. Because – as the reviewer correctly anticipates – we expect to challenge contradictory assignments in two previous papers, we have to be particularly sure that our findings are well substantiated and the description of all experimental evidence will likely exceed letter size. By discovering and presenting the simple and intriguing spectral triad for a series of tertiary amines beyond the elementary prototype (which are also easier to handle for us due to their condensed phase character), the most important message of spectral robustness is conveyed. In this way, we also give a chance to the original authors to reconsider their assignment, before we explicitly dispute it. The VUV-IR studies have been applied to a number of water-containing clusters and published prominently, but we think there is growing evidence that the claimed strict size-selectivity does not apply (we have already indicated this in Ref. (9) and (10) and discussed with a senior author of the VUV-IR studies). This also

adds to the urgency of our present work, because in our opinion, the scientific record should comprise as few as possible incorrect assignments and claims.

- One of the merits of the paper is the reassignment of these previous works. I would have expected more extended developments, in particular :
= for the matrix study, the fact that with trimethylamine a triad is satisfactorily observed at nearly the same locations as in the MMCN molecule.
= for the IR/VUV experiment the absence of a band assignable to a OHb feature and of a triadic pattern as well, is a strong hint at the occurrence of extensive fragmentation processes in the ion, which ruin the claimed mass-selective character of the experiment.

We fully agree with the reviewer, but we have considered it somewhat premature to argue so explicitly on the basis of spectral data for similar, but still different compounds, despite the compelling indirect evidence. This will be done in the next publication on trimethylamine-water, once we have collected enough data for explicit comparison. There, we can also give the criticized authors the opportunity of a statement in the sense of a personal communication, that they agree (or not) with our conclusions.

Beyond the reviewer suggestions, we applied a few minor improvements to the manuscript:

We now elaborate a little more on Fig. 5, by adding:

…but this requires a systematic investigation for several amines. Note that as the temperature goes up and the hydrogen bond weakens, the b2ON combination transition also gains in relative intensity, suggesting that it participates in the intensity stealing from the thermally blue-shifting OHb.

We add a suitable reference on water libration to the corresponding text instance: …will not be a high frequency librational mode [DOI: 10.1039/c8cp05985c]

We further add a compilation of other nitrogen compounds and their hydrates:

…extension to other tertiary amines and further nitrogen compounds

[https://doi.org/10.1063/5.0033071] …

We add the Lot# for 1-azabicyclooctane in the ESI, Tab. S1: DLX952

In Tab. S14 in the ESI, we make small label corrections:

Ohf is changed into OHf

The very weak transition at 3445cm-1 in Tab. S14 is now more cautiously labelled C – this more cautious assignment of the minor peak is carried over to Figs. 1, 4, 5 in the main text. The corresponding caption for Fig. 1 is extended: …from trimers (T), clusters of unclear size (C), and…

In the caption to Table 1, we change “A, B” into “A and B” and we further specify “raw spectroscopic data (dominant peak position)”. We make “0.97ω” more explicit by writing “0.97$\times\omega$”. We leave away the entry “Amine” in the upper left corner of this table, because it could be confusing as spectroscopic constants are listed. We arrange “0” and “-“ for empty entries in a more logical way.

We make the text “…into the water monomer range (M), where the out-of-phase or free OH vibration (OHf) is visible.” clearer by writing ”…into the water monomer stretching range (M), where the out-of-phase or free OH stretch vibration (OHf) is visible.” In this context, “…indicated by the tiny overtone signals” is further specified to “…indicated by the tiny bend overtone signals”

In one instance each, the wording “triple resonance” is clarified as “resonance triad”, and “1:2 Fermi resonance” is replaced by “two-level Fermi resonance”

In Fig. 3, the coupling matrix elements are now italicized for consistency.

For better readability, expressions like “lower frequency…” are changed into “lower-frequency…”

We hope that our manuscript is now suitable for publication in JPC Letters as a first entry point into universal resonance patterns in amine monohydrates.
